# Influencing factors of depressive symptoms among undergraduates: A systematic review and meta-analysis

**DOI:** 10.1371/journal.pone.0279050

**Published:** 2023-03-02

**Authors:** Le Yang, Jiarong Yuan, Huan Sun, Yidan Zhao, Jiajie Yu, Youping Li

**Affiliations:** 1 Chinese Evidence-Based Medicine Center, West China Hospital of Sichuan University, Sichuan, China; 2 West China School of Pharmacy, Sichuan University, Chengdu, China; 3 Mental Health Center, West China Hospital of Sichuan University, Chengdu, China; 4 West China School of Public Health, Sichuan University, Chengdu, China; University of Botswana, BOTSWANA

## Abstract

**Objective:**

This systematic review aims to examine the influencing factors of undergraduates’ depressive symptoms by summarizing the categories and intensity of the factors, to lay a foundation for subsequent research.

**Methods:**

Two authors independently searched in Medline (Ovid), Embase (Ovid), Scopu, PsycINFO, PsycARTICLES, the Chinese Scientific Journal Database (VIP Database), China National Knowledge database (CNKI), and the WanFang database for cohort studies related to the influencing factors affecting depressive symptoms among undergraduates published prior to September 12, 2022. Adjusted Newcastle-Ottawa scale (NOS) was used to assess the risk of bias. Meta-analyses of regression coefficient estimates were performed to calculate pooled estimates with R 4.0.3 software.

**Results:**

A total of 73 cohort studies were included, involving 46362 participants from 11 countries. Factors affecting depressive symptoms were classified into relational, psychological, predictors of response to trauma, occupational, sociodemographic and lifestyle factors. In Meta-analysis, 4 of 7 influencing factors were revealed to be statistically significant: negative coping (B = 0.98, 95%CI: 0.22–1.74), rumination (B = 0.06, 95%CI: 0.01–0.11), stress (OR = 0.22, 95%CI: 0.16–0.28) and childhood abuse (B = 0.42, 95%CI:0.13–0.71). No significant association was found in positive coping, gender and ethnicity.

**Limitations:**

The current studies have the problems of inconsistent use of scales and large heterogeneity of research design, making it difficult to summarize, which is expected to be further improved in future research.

**Conclusion:**

This review evidences the importance of several influencing factors of depressive symptoms among undergraduates. We advocate for more high-quality studies with more coherent and appropriate study designs and outcome measurement approaches in this field.

**Trial registration:**

***Systematic review registration*:** PROSPERO registration CRD42021267841.

## Introduction

Depressive symptoms (also reported as depression), which are typically characterized by sadness, hopelessness, loss of interest, nervous or anxious feelings, and even suicidal ideation, are among the most common health problems in undergraduates [1–4]. Clinical depression is the second leading cause of years lost due to disability (YLDs) and disability-adjusted life years (DALYs) in young people aged 20–24 years [5]. Compared with the general population or noncollege students, college students had higher rates of depressive symptoms, ranging from 24% to 34% [6–10].

Undergraduates are susceptible to depressive symptoms due to their special developmental stage in life, the nature of adjustment to the new environment, and pressure from academic work and/or campus life [6,11,12]. Depressive symptoms could worsen other mental health problems, such as substance abuse, violent behaviors or suicide, resulting in a crippling effect not only on daily life, studying, friendship, and family of undergraduates but also on future employment and work productivity [13].

The number of studies about depressive symptoms in undergraduates has increased rapidly, and most of them have focused on the prevalence. Some published studies exploring influencing factors found that sociodemographic factors (e.g., gender, ethnicity, nationality), psychological factors (e.g., negative coping, attributional style, self-esteem), and negative experience factors (e.g., childhood abuse, negative life events) were associated with depressive symptoms [14–21]. However, the conclusions of these studies may be compromised by methodological flaws, such as small sample size, inconsistent strength and direction of the association, limitation of study design, potential confounders, and/or varied measurement tools. Some specific factors among undergraduates, such as interpersonal relationships and stressors on campus, also need to be considered. Moreover, effective interventions and preventions play an essential role in the development of depressive symptoms, and a better understanding of the potential influencing factors could benefit intervention and prevention.

Therefore, we conducted a comprehensive systematic review and meta-analysis including cohort studies to identify the potentially influential factors of depressive symptoms in undergraduates and explore the strengths of associations.

## Methods

This review was registered on PROSPERO (CRD42021267841) and conducted according to the Preferred Reporting Items for Systematic Reviews and Meta-Analysis (PRISMA) statement guidelines [22] (S1 Table).

### Inclusion and exclusion criteria

Studies eligible for this review were as follows: 1) participants were studying at the undergraduate level without limitations on major, gender, or nationality; 2) depressive symptoms were measured by the five most commonly used scales including BDI (Beck Depression Inventory), SDS (Zung Self-rating Depression Scale), CES-D (Center for Epidemiologic Studies Depression Scale), PHQ-9 (Patient Health Questionnaire-9) and/or BSI (Brief Symptom Inventory); 3) cohort studies with usable outcome data, such as regression coefficient estimates (B), standard error, and 95% confidence intervals; and 4) published in English or Chinese language. If there were multiple publications for the same population, data from the article with the most reliable or useful information were included. We excluded cross-sectional studies, case studies, reliability and/or validity studies, and studies for clinical patients.

### Search strategy

Relevant studies were identified from PubMed, Embase (via Ovid), Scopus, PsycINFO, PsycARTICLES, the Chinese Scientific Journal Database (VIP Database), China National Knowledge database (CNKI), and the WanFang database in July 2021 and updated to September 12, 2022. We use the search terms for depression (“depression”, “depressive disorder”, and “depressive symptom”) combined with terms for undergraduates (“undergraduate student”, “college student”, “university student”) and terms for cohort studies (“cohort studies”, “longitudinal studies”, “retrospective studies”). A highly sensitive search strategy, as consulted with an information expert, was undertaken to optimize our search strategy (S1 File). The reference list of published systematic reviews was cross-checked for additional eligible studies.

### Study selection and data extraction

Two reviewers (L.Y and H.S.) independently used a predefined form to screen the titles, abstracts, and full text of potentially eligible studies. Three reviewers independently assessed the risk of bias and extracted data. If necessary, discrepancies were resolved through discussions. We collected information regarding 1) study characteristics: authorship, year of publication, country, study design, sample size, and follow-up duration; 2) outcome measurement information: measurement times and scales; 3) the influencing factors; and 4) statistical methods and results: linear regression, logistic regression, regression coefficient estimates (B), standard error, and 95% confidence intervals.

### Quality assessment

Two reviewers (L.Y and H.S.) independently assessed the quality using a modified version of the Newcastle-Ottawa Quality Assessment Scale [23,24] (S2 Table). The third reviewer (H.G) reviewed the complete quality assessment, and any disagreements were resolved by consensus. We removed the items “selection of the nonexposed cohort” and “comparability of cohorts based on the design or analysis” for cohort studies without comparison. We also removed the item “demonstration that the outcome of interest was not present at the start” for retrospective single-arm cohort studies. Prospective studies are graded on an ordinal scale with a maximum score of 6 points, and retrospective studies are scored 5 points. Studies having less than 3 points were identified as a high risk of bias.

### Statistical analysis

We summarized and classified the influencing factors according to Furber et al.’s categorization [25]. For each influencing factor, we pooled the data using a random-effect model for potential heterogeneity when more than two studies were reported with regression coefficient estimates. Other factors with insufficient data were listed in a table. Heterogeneity was evaluated using Cochran Q and I^2^ statistics. We expressed coefficient estimates of logistic regression as odds ratios (ORs) with 95% confidence intervals and linear regression as B with 95% CIs. Subgroup analyses were conducted in terms of economic conditions (developed countries versus developing countries), gender (male versus female) and majors (medical students versus other students) when applicable. Sensitivity analysis was performed by sequentially removing each study in turn and reanalyzing the remaining data sets. Publication bias was assessed using Egger test plots when ten or more studies were available. R 4.0.3 software was used for meta-analysis.

## Results

**Fig 1:**
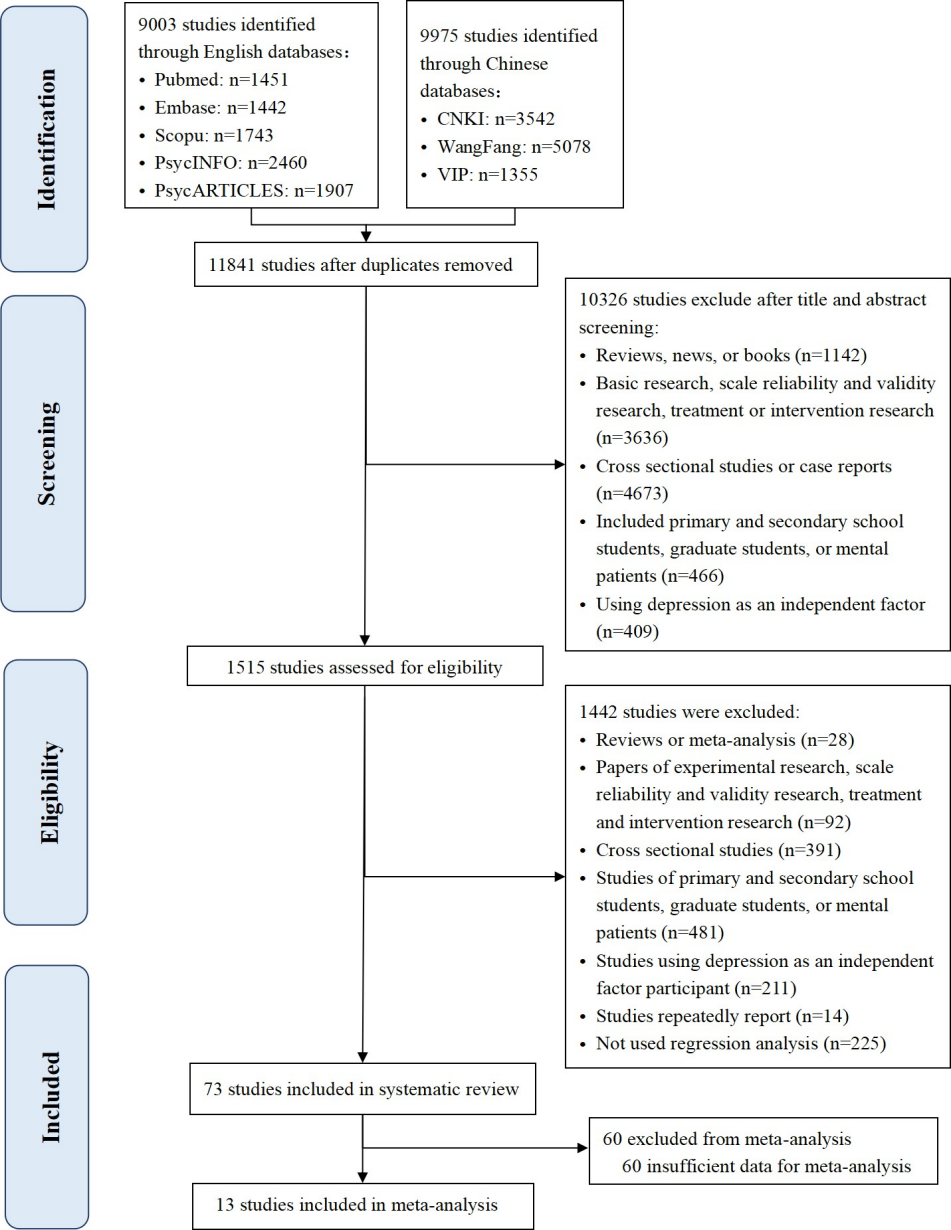
Flowchart of study selection.

Eight databases were screened, yielding a total of 19442 studies. After removing the duplicates and screening the titles and abstracts, 1515 studies were selected for full-length review. Out of these, 1442 that did not meet the inclusion criteria were excluded. Finally, 73 studies involving 46362 undergraduates were included in the systematic review. The process of study selection is listed in Fig 1.

### Characteristics of included studies

Among the 73 cohort studies conducted in 11 countries, 58 (79%) were published in English and 15 (21%) in Chinese. Of these studies, 59 (81%) were prospective cohort studies, and the length of follow-up ranged from 3 weeks to 208 weeks (median 29 weeks). The sample size ranged from 30 to 10340, and the mean age ranged from 17.8 to 22.1 years. Eighteen studies (25%) limited their participants to the first year, and 19 (26%) focused on psychology students. Thirty-six (49%) studies used the BDI tool to measure depressive symptoms, followed by the CES-D (n = 18, 25%) and SDS scale (n = 11, 15%) (Tables 1 and S3). Study quality varied among six items and 17 studies (23.3%) were at high risk of bias (S4 Table).

**Table 1:** The characteristics of included studies.

		Number of studies	%
**Total number of studies**		73	100
**Language**	English	58	79.5
	Chinese	15	20.6
**Study design**	Prospective cohort	59	80.8
	Retrospective cohort	14	19.2
**Length of follow-up of prospective cohort**	≤3 months	27	37.0
	4–6 months	16	21.9
	7–12 months	10	13.7
	>12 months	6	8.2
**Cohort size (participants)**	n <100	12	16.4
	100~499	44	60.3
	500~999	7	9.6
	1000~9999	9	12.3
	n>10000	1	1.4
**Region (countries)**	US	31	42.5
	China	21	28.8
	Japan	6	8.2
	Other countries	15	20.6
**Grade of participants**	Freshmen	18	24.7
	Other grades*	19	25.7
	Multiple grades^&^	11	14.9
	Not reported	25	33.8
**Major of participants**	Psychology	19	25.7
	Medical	4	5.4
	Science and Technology	1	1.4
	Sociology and Humanities	2	2.7
	Multiple majors	4	5.4
	Not reported	43	59.5
**Use of depressive symptoms scales**	BDI	36	48.7
	CES-D	18	24.3
	SDS	11	14.9
	PHQ-9	5	6.8
	BSI	3	4.1

* Other grades: Any grade other than freshmen (i.e. sophomore, junior, or senior).

^&^ Multiple grades: More than one grade.

### The influencing factors related to depressive symptoms

We identified 70 influencing factors of depressive symptoms and summarized them into six categories according to Furber et al.’s category model [25], including relational, psychological, predictors of response to trauma, occupational, sociodemographic and lifestyle (Table 2).

**Table 2:** Influencing factors of depressive symptoms in 73 studies.

Primary category	Secondary category	Reported influencing factors	No of studies	NOS score of studies*
Relational	Parent/caregiver-child relationships	***Family support***	2	5
		**Childhood abuse**	8	4
		**Insecure attachment**	4	4
		**Poor parental style**	1	4
		**Role reversal-mother**	1	4.5
	Social and peer support	***Social support; Social satisfaction; Social intimacy***	7	4
		**Social inhibition; loneliness**	2	4.5
		**Bullying victimization**	1	4
		**Partner-initiated breakup**	1	4
		**Sexual assault**	1	5
Psychological	Personality	***Self-worth; Self-esteem***	5	4
		***Optimism***	1	5
		**Perfectionism; Self-oriented perfectionism; Socially prescribed perfectionism; Other perfectionism**	4	4
		**Masculinity**	1	4
		**Neuroticism**	2	4.5
		**Orality**	1	4
		*Autonomy*	1	4
	Positive coping styles	**Positive coping; Seeking social support; Adaptive emotion regulation**	4	5
		*Distractive response*	1	4
	Negative coping styles	**Negative coping; Avoidance coping; Self-preoccupation; Maladaptive emotion regulation; Catastrophizing emotion regulation; Self-criticism**	9	4
		**Rumination; Brooding; Reflection**	7	4
		*External preoccupation*	1	3
	Interpersonal-related cognitive factors	**Emotional deprivation; Neediness; Connectedness; Excessive reassurance seeking**	5	5
		*Rejection sensitivity; Abandonment; Defectiveness*	3	5
	Other cognitive factors	**Generalization**	2	3
		**Negative Cognitive Style**	2	3.5
		**Automatic negative thoughts**	2	4
		**Dysfunctional Attitudes**	2	3
		**Validation seeking**	1	4
		**Hopelessness**	1	3
		***Psychological resilience***	*1*	*5*
		*Attributional Style*	2	2.5
		*High Standards*	1	2
		*Failure*	1	5
Predictors of response to trauma		**Negative Life Events; Frequency of current problems**	22	5
		**Academic stress events; Occurrence of a midterm or major assignment**	3	5
		**Ill health and adaptation events**	2	5
		**Punishment events**	1	5
		**Loss events**	1	5
		**Interpersonal events**	1	5
		**Perceived stress**	1	5
Sociodemographic	Non-modifiable sociodemographic factors	**Female**	3	4
		**Ethnicity (African American; Hispanic/Latino)**	2	4
		**Sexual minority**	1	3
Occupational	Work environment	**Sexual harassment**	1	3
Lifestyle		**High alcohol quantity**	1	5
		*Exercise frequency*	1	5

Bolded: Significant risk factors.

bolded and italicized: Significant protective factors.

italicized: No association.

* If there are more than one paper in this category, the median of their NOSs is provided.

#### Relational

A total of 23 studies have explored the relationship between relational factors and depressive symptoms of undergraduates.

*Parent/Caregiver-child relationships*. Childhood abuse was a significant risk factor for depressive symptoms across eight studies, and insecure attachment to parents, poor parental style and role reversal with their mother also increased the risk of depression in adulthood. High levels of family support were identified as a positive factor in depression.

*Social and peer support*. Social support, including social satisfaction and social intimacy, was consistently found to reduce the risk of depressive symptoms. Undergraduates who suffered a partner-initiated breakup, sexual assault, sexual harassment, bullying victimization, and/or felt social inhibition or loneliness were susceptible to depressive symptoms.

#### Psychological

Psychological factors in undergraduates were widely discussed in 42 studies, which were grouped into personality, coping, interpersonal cognitive style and other cognitive factors.

*Personality*. Five studies have explored the relationship between self-worth (self-esteem) and depressive symptoms, with one of them showing that self-worth could decrease the risk of depressive symptoms. Two studies reported that perfectionism and its sub dimensions (i.e., self-oriented perfectionism and socially prescribed perfectionism) were positively related to depressive symptoms. Meanwhile, neuroticism, orality, masculinity and optimism also positively influenced depressive symptoms.

*Positive coping styles*. Five studies reported the effect of positive coping styles and showed that positive coping, seeking social support and adaptive emotion regulation were negatively related to depressive symptoms; however, this correlation was not found in distractive responses.

*Negative coping styles*. The relationship between rumination (ruminative coping) and depressive symptoms was inconsistent among seven studies. However, brooding and reflection were identified as positive factors of depressive symptoms. Other negative coping styles (i.e., negative coping, avoidance coping, self-preoccupation, maladaptive emotion regulation, catastrophizing emotion regulation, and self-criticism) were associated with depressive symptoms, except external preoccupation.

*Interpersonal-related cognitive factors*. Eight studies suggested the association between depressive symptoms and emotional deprivation, neediness, connectedness and excessive reassurance-seeking.

*Other cognitive factors*. Generalization, negative cognitive style (total score of CSQ scale), dysfunctional attitudes, validation seeking, hopelessness and psychological resilience were consistently found to be related to depressive symptoms.

#### Predictors of response to trauma

Twenty-nine studies reported the relationship between predictors of response to trauma and depressive symptoms of undergraduates. Suffering from negative life events was an important risk factor for depressive problems, which was reported in 21 studies. Stressful events that predicted major depression included academic stress events, interpersonal events, punishment events, loss events, and health and adaptive stress. Perceived stress and the frequency of current problems also were predictors of depressive symptoms.

### Other factors

We summarized sociodemographic factors, occupational factors and lifestyle in other factors. Three studies consistently showed a significant association between gender (female) and depressive symptoms. Two studies showed that ethnicity was related to depressive symptoms. One study identified sexual harassment as an influencing factor in depressive symptoms, and alcohol quantity is also a significant predictor of depressive symptoms.

### Meta-analysis of influencing factors

**Fig 2:**
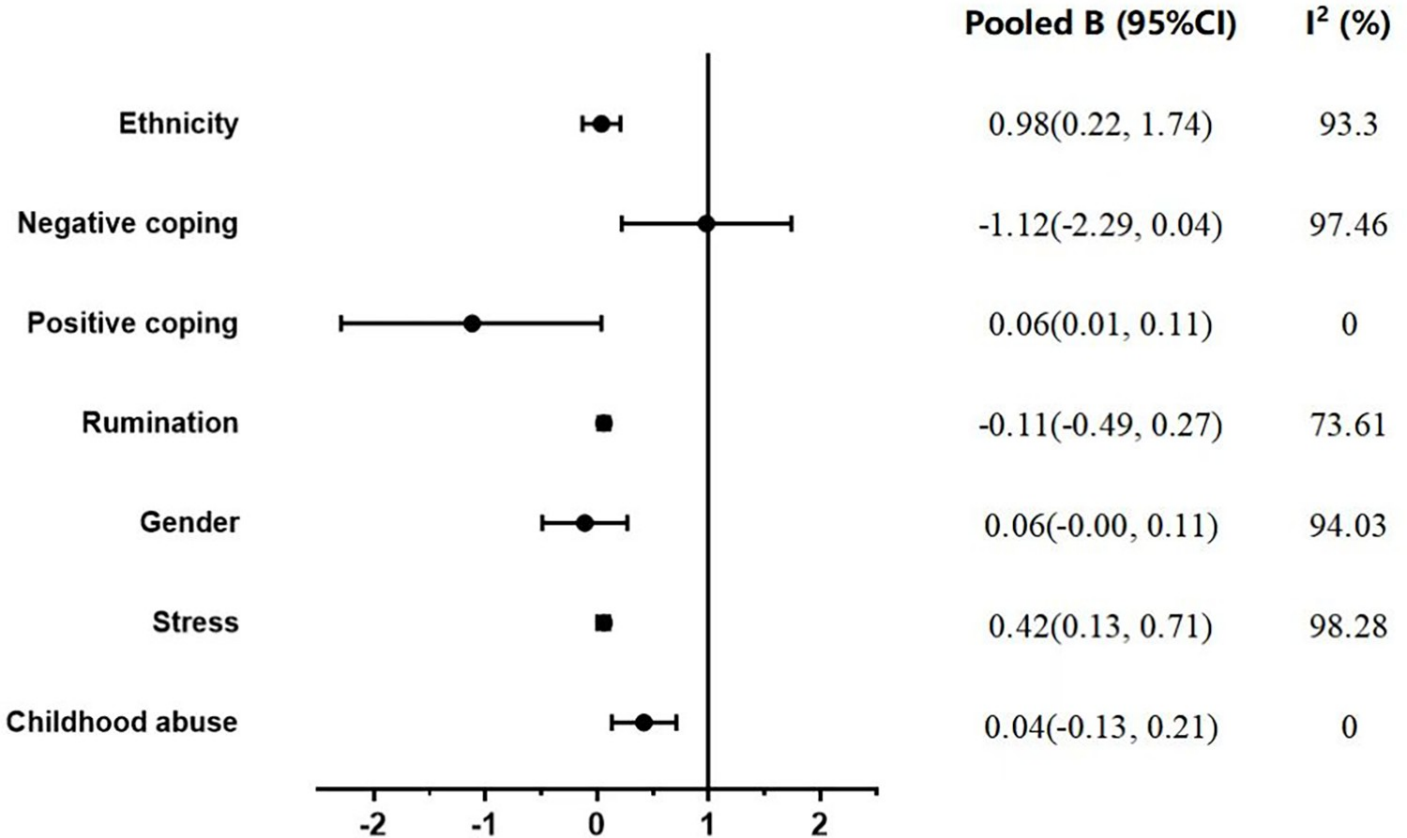
Summary of meta-analysis results.

Thirteen studies involving 21642 undergraduates contributed to meta-analytic comparisons of 7 influencing factors across the depression. The pooling data of these studies showed that negative coping (B = 0.98, 95%CI: 0.22–1.74), rumination (B = 0.06, 95%CI: 0.01–0.11), stress (OR = 0.22, 95%CI: 0.16–0.28) and childhood abuse (B = 0.42, 95%CI:0.13–0.71) were significantly associated with depression (Figs 2 and S1). However, no significant association was found in positive coping, gender and ethnicity. Other influencing factors with OR values less than two are listed in Table 2.

We did not conduct the subgroup analysis and assess publication bias for the small number of studies included in each factor.

## Discussion

This review brings all cohort studies together to explore the influencing factors of depressive symptoms among undergraduates. Both the narrative synthesis and meta-analysis highlight a range of influencing factors, including psychological, relational, response to trauma, occupational, lifestyle and demographic factors. Specifically, the meta-analyses showed that four risk factors, i.e., negative coping, rumination, stress and childhood abuse, were significantly associated with depression.

### Relational

Two important relational factors were identified in the narrative synthesis: childhood abuse and poor social support. The meta-analysis results found childhood abuse to be a significant predictor of depression, but social support was not analyzed by meta-analysis due to the inadequate reporting of the nonstandard regression coefficient. Childhood abuse, which includes sexual abuse, physical abuse, emotional abuse, emotional neglect, and physical neglect, has a wide range of influences on an individual’s cognition, emotion, personality, coping styles, behavior, self-consciousness and social functions, resulting in depression [19,26–28]. Childhood abuse experience represents a nonmodifiable risk factor that helps universities to identify high-risk undergraduates. Most of the included studies were conducted in China (6 of 8), indicating that Chinese researchers have paid great attention to child abuse problems in recent years.

The effects of increased social support on reducing distress have been strongly evidenced in previous studies [29]. In our study, six studies reported that social support predicted follow-up depressive symptoms after controlling for demographic factors and baseline depressive symptoms, which is consistent with the literature [30–32]. To improve social support may therefore be an effective intervention, because it is a factor that can be changed.

### Psychological

Among the psychological factors, two key risk factors were identified in the narrative synthesis and meta-analysis: negative coping style and rumination. The latter was further categorized into brooding and reflecting. According to the diathesis-stress model, many studies reported that the negative coping style had an effect on the subsequent depression when triggered by the presence of stressors [30,33,34]. Negative coping aims at escaping the stressor(s) or related emotions and includes strategies such as denial, avoidance, and fantasy. These strategies are generally ineffective in reducing stress over time, as they ignore the stressor’s existence and its eventual consequences, leading to increased stress (acute and chronic) and subsequent increases in depressive symptoms [34].

Rumination is one of the negative coping styles, which means “repetitively focusing on the fact that one is depressed; on one’s symptoms of depression; and on the causes, meanings, and consequences of depressive symptoms” [35], so it is conceptualized as an emotion regulation strategy or a meta-emotional cognitive process [31].

### Predictors of response to trauma

Most studies on trauma factors focus on a total score of daily negative life events (22 studies, most of which are high quality), and 20 of these studies showed a strong correlation between negative life events and depressive symptoms. Among the specific types of negative life events, studies have proven academic stress events and interpersonal events. punishment events, loss events, health and adaptation events can predict depressive symptoms [36,37]. Only a few articles of specific events are included in our study, but they can provide strong evidence due to their high research quality.

To rate the impact of daily life events, many studies used questions to measure negative life stress, such as “Consider events you experienced during the past week which were undesirable, upsetting, or which made you unhappy or sad” [12], or “Within the last 12 months, how would you rate the overall level of stress you have experienced?” [14]. There was a strong correlation between perceived stress and subsequent depressive symptoms, the regression analysis conclusion was consistently positive between studies, and the meta-analysis showed a statistically significant effect. Perceived stress refers to the stress felt in the face of events beyond one’s ability to cope with. It is more strongly correlated with depressive symptoms than the negative event itself and affects the subsequent depressive level after controlling for age, gender, and baseline depression [38,39].

### Other factors

The narrative summary reported female undergraduates as a particularly high-risk group, although the results were not significant in the meta-analysis. This is consistent with the literature reporting a greater proportion of females with common mental health difficulties [40,41]. Some studies have shown that ethnicity and sexual orientation are related to depressive symptoms. Minorities such as African Americans and homosexuality are more vulnerable to depressive symptoms. This suggests that these people may face less social support and worse economic and social status, so they bear more pressure from peers and daily life [14,15].

### Limitations and strengths

We conducted a comprehensive systematic review including all cohort studies with rigorous methods to explore the potential influencing factors of depressive symptoms. Our review provided a full picture of the multidimensional influencing factors in 73 included articles and combined some of the factors with B(SE) or ORs (95% CI). We excluded students in clinical populations and may therefore reflect the situation of general college students. Given the number of cohort studies screened during study selection, the review has wide-reaching coverage of relevant research. Excluding low-quality cross-sectional designs also allowed for definitive cause-and-effect relationships to be determined.

Nevertheless, our study has a few limitations. First, our review yielded a respectable number of articles, and the field of influencing factors is largely heterogeneous. The varied quality of studies, length of follow-up and reporting of statistical findings led to the small number of homogeneous studies and limited the extent to which we were able to statistically synthesize the datasets. Importantly, the scales used to measure influencing factors were inconsistent. The differences in how influencing factors were defined therefore limited the number of articles eligible for risk factor meta-analysis. Second, we were unable to conduct a subgroup analysis to explore the source of heterogeneity for a limited number of studies. Third, we used a validated risk categorization scheme developed by Furber et al. for the narrative synthesis, which covered a diverse range of predictors for mental illness. However, the framework was not developed for undergraduates. Especially, categories such as “work environment” do not adequately reflect the pressures in college and may therefore be inappropriate. Finally, publishing biases have been introduced due to the unavailability of databases, nonpublished data, offline studies and papers published in languages other than English and Chinese.

## Conclusion and future directions

In summary, our findings show multiple and complex influencing factors of depressive symptoms among undergraduates, including psychological, relational, stress and demographic factors. Early recognition of at-risk students presents an opportunity to prevent depression by intervention early in the university experience. Our research can help universities, health systems and policymakers to identify groups of undergraduates at risk of depression, which is an essential condition for targeting effective preventive care.

The current studies have the problems of inconsistent use of scales, large heterogeneity of research design, and limited information about regression coefficient and/or SE, making it difficult to summarize, which is expected to be further improved in future research. We also advocate for more high-quality studies in this field, including more coherent and appropriate study designs and outcome measurement approaches, based on the methodological problems, between-study heterogeneity and inconsistent terminology highlighted in this review, and more accurate reports of the parameters so that they can be statistically summarized.

## Supporting information

S1 Fig Forest plots of the meta-analysis. (TIF)

S1 Table PRISMA checklist. (DOCX)

S2 Table The modified version of Newcastle-Ottawa scale. (DOCX)

S3 Table Characteristics of the included studies. (DOCX)

S4 Table Literature quality evaluation. (DOCX)

S1 File Search strategy of the research. (DOCX)
