# Supplementary figures and images for: Influencing factors of depressive symptoms among undergraduates: A systematic review and meta-analysis
Source: PLoS One. 2023 Mar 2;18(3):e0279050. doi: 10.1371/journal.pone.0279050 (PMC9980735; doi:10.1371/journal.pone.0279050)

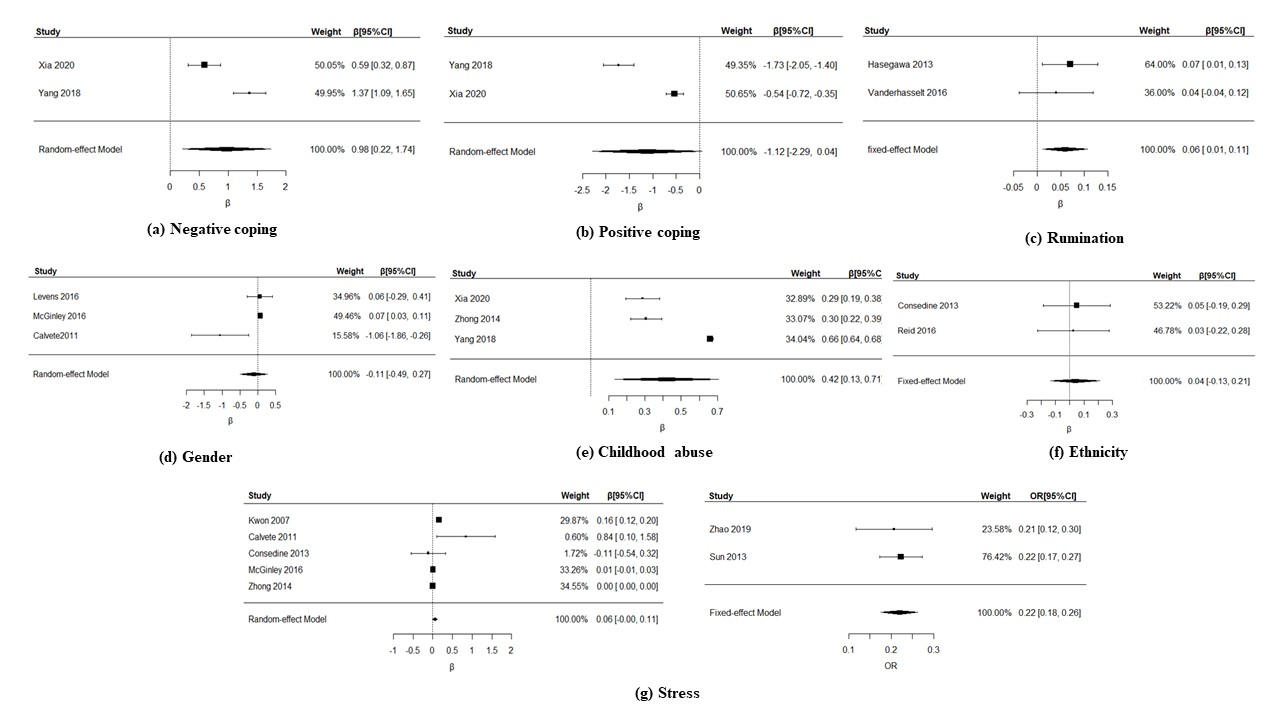

Supplement: S1 Fig — (TIF) [file pone.0279050.s001.tif]
