# Supplementary material for: Influencing factors of depressive symptoms among undergraduates: A systematic review and meta-analysis
Source: PLoS One. 2023 Mar 2;18(3):e0279050. doi: 10.1371/journal.pone.0279050 (PMC9980735; doi:10.1371/journal.pone.0279050)
Supplement: S2 Table — (DOCX) [file pone.0279050.s003.docx]

**Supporting information S2 Table. The modified version of Newcastle-Ottawa scale**

| NOS Checklist |  | Modified NOS Checklist and the scoring basis |
| --- | --- | --- |
|  |  |  |
| **Selection** |  | **Selection** |
| 1.Representativeness of the exposed cohort |  | 1.Representativeness of the cohort |
| a) truly representative of the average_ (describe) in the community ♦ |  | 1. randomized sampling♦ |
| b) somewhat representative of the average in the community ♦ |  | b) non-specific types of undergraduates who are somewhat representative ♦ |
| c) selected group of users e.g., nurses, volunteers |  | c)students and volunteers in psychology courses |
| d) no description of the derivation of the cohort |  | d)no description of the derivation of the cohort |
| 2. Selection of the non-exposed cohort |  |  |
| a) drawn from the same community as the exposed cohort ♦ |  |  |
| b) drawn from a different source |  |  |
| c) no description of the derivation of the non-exposed cohort |  |  |
| 3. Ascertainment of exposure |  | 2. Ascertainment of exposure |
| a) secure record (e.g., surgical records) ♦ |  | a) objective records such as credits♦ |
| b) structured interview ♦ |  | b) measured by recognized scales or self-made scales with good reliability and validity ♦ |
| c) written self-report |  | c) self-report of non-scale measurement |
| d) no description |  | d) no description |
| 4. Demonstration that outcome of interest was not present at start of study |  | 3. Demonstration that outcome of interest was not present at start of study(only for prospective studies) |
| a) yes ♦ |  | a. yes ♦： 1) The baseline depressed population is excluded; or 2) the baseline depressed population is not excluded, but controlled during statistics, or the difference between measurements was used as the outcome indicator for analysis. |
| b) no |  | b) no (the baseline depressed population is not excluded, and the influences of the baseline depression is not considered during statistics.) |
| **Comparability** |  |  |
| 5. Comparability of cohorts on the basis of the design or analysis |  |  |
| a) study controls for (select the most important factor) ♦ |  |  |
| b) study controls for any additional factor (This criterion could be modified to indicate specific control for a second important factor.) ♦ |  |  |
| **Outcome** |  | **Outcome** |
| 6. Assessment of outcome |  | 4. Assessment of outcome |
| a) independent blind assessment ♦ |  | a)Clinical diagnosis and/or a recognized depression scale ♦ |
| b) record linkage ♦ |  | b) a self-made scale with good reliability and validity ♦ |
| c) self-report |  | c) self-report of non-scale measurement |
| d) no description |  | d) no description |
| 7. Was follow up long enough for outcomes to occur |  | 5. Was follow up long enough for outcomes to occur |
| a) yes (select an adequate follow up period for outcome of interest) ♦ |  | a) ≥3 months ♦ |
| b) no |  | b) <3 months |
| 8. Adequacy of follow up of cohorts |  | 6. Adequacy of follow up of cohorts |
| a) complete follow up - all subjects accounted for ♦ |  | a) complete follow up ♦ |
| b) subjects lost to follow up unlikely to introduce bias - small number lost - > ___ % (select an adequate %) follow up, or description of those lost) ♦ |  | b) subjects lost to follow up unlikely to introduce bias - follow up rate ≥80% or slightly < 80%, but reporting no heterogeneity between the follow-up population and loss population. ♦ |
| c) follow up rate < ___% (select an adequate %) and no description of those lost |  | c) the follow-up rate was < 80% and no description of those lost |
| d) no statement |  | d) no statement |
