# Supplementary material for: Influencing factors of depressive symptoms among undergraduates: A systematic review and meta-analysis
Source: PLoS One. 2023 Mar 2;18(3):e0279050. doi: 10.1371/journal.pone.0279050 (PMC9980735; doi:10.1371/journal.pone.0279050)
Supplement: S3 Table — (DOCX) [file pone.0279050.s004.docx]

**Supporting information S3 Table. Characteristics of the included studies**

| NO | Study | Country | Design | Follow-up length | Sample Characteristics | | | | | Measure of depressive symptoms | NOS score |
| --- | --- | --- | --- | --- | --- | --- | --- | --- | --- | --- | --- |
|  |  |  |  |  | N | Grade | Age | Sex (M/F) | Major |  |  |
| 1 | Nezu1986 | USA | Prospective | 8W | 129 | Other grades | 22.1±2.64（18-25） | 81/48 | Multiple majors | BDI | 3 |
| 2 | Klocek1997 | USA | Prospective | 10W | 266 | Other grades | Not reported | Not reported | Psychology | BDI | 2 |
| 3 | Carver1998 | USA | Prospective | 6W | 336 | Other grades | Not reported | 151/185 | Multiple majors | BDI | 2 |
| 4 | Dykman1998 | USA | Prospective | 14W | 300 | Other grades | Not reported | 119/181 | Psychology | BDI | 4 |
| 5 | Gershuny1998 | USA | Prospective | 156W | 489 | Freshmen | 18.2±0.96 | Not reported | Not reported | BSI | 5 |
| 6 | Kapci1998 | England | Prospective | 13W | 80 | Multiple grades | Not reported | Not reported | Not reported | BDI | 4 |
| 7 | Cheng1999 | China | Prospective | 27W | 175 | Other grades | Not reported | 75/103 | Psychology | BDI | 4 |
| 8 | Morris1999 | Australia | Prospective | 45W | 363 | Freshmen | 22.04 | 108/245 | Psychology | BDI | 3 |
| 9 | Sakamoto1999 | Japan | Prospective | 13W | 284 | Multiply grades | Not reported | 222/62 | Psychology | SDS | 4 |
| 10 | Sakamoto2000 | Japan | Prospective | 18W | 284 | Multiply grades | Not reported | 222/62 | Psychology | SDS | 3 |
| 11 | Ayduk2001 | USA | Prospective | 52W | 223 | Freshmen | 18.5±0.57 | 0/223 | Not reported | BDI | 4 |
| 12 | Davila2001 | USA | Prospective | 27W | 94 | Freshmen | 18.05±0.31 | 49/45 | Not reported | BDI | 6 |
| 13 | Enns2001 | Canada | Prospective | 27W | 96 | Other grades | 25.1(20-48) | 56/40 | Medical | BDI | 5 |
|  |  |  |  |  | 289 | Freshmen | 19.1(17-50) | 106/183 | Psychology |  |  |
| 14 | Fresco2001 | USA | Prospective | 8W | 78 | Not reported | 20±3.7 | 24/54 | Psychology | BDI | 3 |
| 15 | Smith2001 | England | Prospective | 18W | 35 | Not reported | Not reported | 24/11 | Not reported | BDI | 4 |
|  |  |  |  |  | 30 | Other grades | Not reported | 20/10 |  |  |  |
| 16 | Scheier2002 | USA | Prospective | 16W | 99 | Freshmen | Not reported | Not reported | Not reported | BDI | 5 |
| 17 | Abela2004 | Canada | Prospective | 5W | 165 | Other grades | Not reported | 57/108 | Psychology | BDI | 4 |
| 18 | Liu2004 | Japan | Prospective | 3W | 437 | Multiply grades | Not reported | Not reported | Not reported | SDS | 3 |
| 19 | Shahar2004 | USA | Prospective | 5W | 207 | Not reported | Not reported | Not reported | Psychology | BDI | 3 |
| 20 | Lindsay2005 | USA | Prospective | 3W | 152 | Not reported | Not reported | Not reported | Psychology | BDI | 4 |
| 21 | Morrison2005 | England | Prospective | 27W | 249 | Not reported | Not reported | Not reported | Not reported | CES-D | 5 |
| 22 | Wingate2005 | USA | Prospective | 5W | 169 | Other grades | 19 | 78/91 | Psychology | BDI | 3 |
| 23 | Joiner2006 | USA | Prospective | 3W | 143 | Not reported | Not reported | 51/92 | Psychology | BDI | 4 |
| 24 | Luhtanen2006 | USA | Prospective | 27W | 795 | Freshmen | 17.78±0.66（16-22） | 343/451 | Not reported | CES-D | 5 |
| 25 | Haeffel2007 | USA | Prospective | 5W | 261 | Not reported | Not reported | Not reported | Psychology | BDI | 4 |
| 26 | Ito2007 | Japan | Prospective | 36W | 346 | Not reported | Not reported | 174/172 | Sociology and Humanities | SDS | 4 |
| 27 | Kwon2007 | USA | Prospective | 4W | 388 | Not reported | Not reported | Not reported | Multiple majors | BDI | 4 |
| 28 | Bjornsson2010 | USA | Prospective | 12W | 748 | Not reported | 18.81±1.17 | 279/469 | Psychology | BDI | 2 |
| 29 | Haeffel2010 | USA | Prospective | 5W | 261 | Not reported | Not reported | Not reported | Not reported | BDI | 5 |
| 30 | Calvete2011 | Spain | Prospective | 22W | 510 | Multiple grades | 19.16±1.69 | 179/331 | Science and Technology, Social and Humanity | CES-D | 5 |
| 31 | Chang2011 | USA | Prospective | 9W | 177 | Other grades | 19.7 (18-23) | 42/135 | Psychology | BDI | 3 |
|  |  | Japan | Prospective |  | 155 | Other grades | 20(18-23) | 71/84 | Psychology |  |  |
| 32 | Haeffel2011 | USA | Prospective | 4W | 131 | Other grades | Not reported | Not reported | Psychology | BDI | 5 |
| 33 | Zou2011 | China | Prospective | 4W | 668 | Not reported | 20.14±1.14 | 310/337 | Not reported | CES-D | 4 |
| 34 | Boujut2012 | France | Prospective | 27W | 1100 | Not reported | 18.8±1.4 | 400/700 | Not reported | BDI | 4 |
| 35 | Goldring2012 | USA | Prospective | 11W | 37 | Not reported | 19-21 | 19/18 | Not reported | CES-D | 4 |
| 36 | Huang2012 | China | Prospective | 13W | 261 | Other grades | Not reported | Not reported | Not reported | BDI | 5 |
| 37 | Reilly2012 | USA | Prospective | 6W | 390 | Not reported | Not reported | Not reported | Psychology | BDI | 2 |
| 38 | Consedine2013 | USA | Prospective | 36W | 305 | Freshmen | Not reported | Not reported | Not reported | CES-D | 4 |
| 39 | Hasegawa2013 | Japan | Prospective | 8W | 437 | Not reported | 19.3 | 196/241 | Psychology | CES-D | 4 |
| 40 | Sun2013 | China | Prospective | 52W | 10340 | Not reported | Not reported | Not reported | Medical | BDI | 5 |
| 41 | Takagishi2013 | Japan | Prospective | 3W | 407 | Not reported | Not reported | Not reported | Medical | SDS | 3 |
| 42 | Zhang2013 | China | Prospective | 18W | 206 | Other grades | Not reported | 97/109 | Not reported | BDI | 4 |
| 43 | Campos2014 | Portugal | Prospective | 27W | 81 | Other grades | Not reported | 0/81 | Psychology | CES-D | 4 |
| 44 | He2014 | China | Prospective | 27W | 1427 | Multiple grades | 20.01±0.99 | 627/587 | Not reported | PHQ-9 | 4 |
| 45 | Ling2014 | China | Prospective | 6W | 900 | Multiple grades | 19.59±1.86(17-24) | 423/401 | Not reported | CES-D | 3 |
| 46 | Pfeifer2014 | USA | Prospective | 104W | 171 | Other grades | Not reported | Not reported | Not reported | BDI | 3 |
| 47 | Vanderhasselt2014 | Belgium | Prospective | 13W | 92 | Other grades | 20.27±2.04 | 20/72 | Not reported | BSI | 5 |
| 48 | Zheng2014 | China | Prospective | 32W | 659 | Multiple grades | 20.11±1.101(16-23) | 304/355 | Not reported | CES-D | 5 |
| 49 | Levens2016 | USA | Prospective | 12W | 181 | Not reported | 18.3±0.91(18-29) | 145/36 | Not reported | CES-D | 4 |
| 50 | McGinley2016 | USA | Prospective | 156W | 2984 | Multiple grades | Not reported | 1191/1664 | Not reported | CES-D | 3 |
| 51 | Reid2016 | USA | Prospective | 27W | 1474 | Freshmen | Not reported | 513/961 | Not reported | PHQ-9 | 4 |
| 52 | Vanderhasselt2016 | Belgium | Prospective | 67W | 92 | Other grades | 20.27±2.04 | 20/72 | Not reported | BDI | 4 |
| 53 | Junkins2017  study-1 | USA | Prospective | 5W | 160 | Not reported | 19.33 | 57/103 | Psychology | BDI | 5 |
|  | Junkins2017  study-2 | USA | Prospective | 27W | 216 | Freshmen | 18 | Not reported | Not reported |  |  |
| 54 | Richardson2017 | England | Prospective | 61W | 454 | Freshmen | 19.9 | 100/352 | Not reported | CES-D | 4 |
| 55 | Kate2018 | USA | Prospective | 18W | 483 | Freshmen | not reported | 0/483 | Not reported | PHQ-9 | 5 |
| 56 | Cheung2020 | China | Prospective | 36W | 154 | Other grades | 20.10±2.13(18-27) | 0/154 | Not reported | PHQ-9 | 4 |
| 57 | Wang2020 | China | Prospective | 18W | 1428 | Freshmen | 18.8±0.9 | 555/873 | Not reported | BDI | 3 |
| 58 | Wu2020 | China | Prospective | 52W | 1064 | Not reported | 18.23±0.76(17-21) | not reported | Not reported | CES-D | 5 |
| 59 | Samek2022 | USA | Prospective | 208W | 209 | Freshmen | 19.1±0.41(18.2-20.1) | 79/103 | Not reported | CES-D | 6 |
| 60 | Katz2009 | USA | Retrospective | \ | 163 | Not reported | Not reported | 0/163 | Not reported | CES-D | 4 |
| 61 | Stolow2012 | Canada | Retrospective | \ | 60 | Other grades | 21.1 | 11/49 | Psychology | BDI | 4 |
| 62 | Zhong2014 | China | Retrospective | \ | 569 | Multiple grades | 21.48±1.58 | 237/298 | Not reported | CES-D | 5 |
| 63 | He2016 | China | Retrospective | \ | 312 | Freshmen | 19.6±3（17-29） | 172/140 | Not reported | SDS | 5 |
| 64 | Adjorlolo2017 | Ghana | Retrospective | \ | 300 | Not reported | 20±2.77 | 94/206 | Not reported | BDI | 5 |
| 65 | Hu2018 | China | Retrospective | \ | 262 | Multiple grades | 19.9±0.9（17-22） | 132/130 | Medical | BDI | 4 |
| 66 | Li2018 | China | Retrospective | \ | 2346 | Freshmen | not reported | not reported | Not reported | SDS | 5 |
| 67 | Rong2018 | China | Retrospective | \ | 485 | Freshmen | 18.83（18-22） | 0/485 | Multiple majors | SDS | 5 |
| 68 | Yang2018 | China | Retrospective | \ | 1802 | Freshmen | 19.49±0.82 | 229/1573 | Not reported | SDS | 5 |
| 69 | Fasciano2019 | USA | Retrospective | \ | 90 | Not reported | 19.21±1.21(18-24) | 27/63 | Not reported | BSI | 5 |
| 70 | Zhao2019 | China | Retrospective | \ | 213 | Multiple grades | not reported | 102/111 | Medical | SDS | 5 |
| 71 | Ma2020 | China | Retrospective | \ | 3609 | Not reported | 20.30±1.23 | 1771/1838 | Not reported | CES-D | 5 |
| 72 | Wang2020 | China | Retrospective | \ | 404 | Not reported | 20.12±1.39 | 189 / 215 | Not reported | PHQ-9 | 5 |
| 73 | Xia2020 | China | Retrospective | \ | 423 | Not reported | 20.89±1.20 | 400/23 | Sociology and Humanity | SDS | 5 |
