# Supplementary material for: Influencing factors of depressive symptoms among undergraduates: A systematic review and meta-analysis
Source: PLoS One. 2023 Mar 2;18(3):e0279050. doi: 10.1371/journal.pone.0279050 (PMC9980735; doi:10.1371/journal.pone.0279050)
Supplement: S4 Table — (DOCX) [file pone.0279050.s005.docx]

**Supporting information S4 Table. Literature quality evaluation**

**Supporting information S4-1. Literature quality evaluation of prospective cohort studies**

Nezu1986

| Item | Description | Judgement | Score |
| --- | --- | --- | --- |
| Item1 | Students were enrolled in various classes at a northeastern university. | b | 1 |
| Item2 | 1.LES:Life Experiences Survey；2.PCL:a modified version  of the Problem Check List | b | 1 |
| Item3 | Not reported | b | 0 |
| Item4 | 21 items to assess depressive symptomatology; reliability and validity are 0 .78 and 0.77. | a | 1 |
| Item5 | 8 Weeks | b | 0 |
| Item6 | Not reported | d | 0 |

Klocek1997

| Item | Description | Judgement | Score |
| --- | --- | --- | --- |
| Item1 | Recruited from introductory psychology classes at Saint Louis University and offered extra class credit for their participation in a study of emotion. | c | 0 |
| Item2 | Dysfunctional Attitudes Scale-Form A (DAS) (Weissman, 1980) | b | 1 |
| Item3 | To remove the influence of initial symptom levels, T1 BDI was entered into the regression first. | b | 0 |
| Item4 | The Beck Depression Inventory (Beck, Ward, Mendelson, Mock, & Erbaugh, 1961) | a | 1 |
| Item5 | 10W | b | 0 |
| Item6 | 74% | c | 0 |

Carver1998

| Item | Description | Judgement | Score |
| --- | --- | --- | --- |
| Item1 | Participants were 336 undergraduates from the University of Miami (151 men, 185 women), who completed the measures Description below in partial fulfillment of a course requirement. | c | 0 |
| Item2 | Attitudes Toward Self-Revised, or ATS-R, High Standards α=0.76 ，Self-Criticism α= 0.78 , Generalization α=0.78 | b | 1 |
| Item3 | Controlling for BDI at Time 1 | b | 0 |
| Item4 | BDI(the short form of the Beck Depression Inventory), 13 items | a | 1 |
| Item5 | 6W | b | 0 |
| Item6 | Not reported | d | 0 |

Dykman1998

| Item | Description | Judgement | Score |
| --- | --- | --- | --- |
| Item1 | College students enrolled in introductory psychology who received course credit for their participation. we divided subjects into two groups‚ an initially asymptomatic group (T1 BDI ≤ 9) and an initially symptomatic group (T1 BDI ≥ 10). | c | 0 |
| Item2 | Dysfunctional Attitudes Scale (DAS‚ Form A; Weissman‚ 1979) | a | 1 |
| Item3 | To control for baseline levels of dysphoria‚ T1 BDI was the first variable entered into the regression equation in each analysis reported. | b | 0 |
| Item4 | Beck Depression Inventory (BDI; Beck‚ Rush‚ Shaw‚& Emery‚ 1979) | a | 1 |
| Item5 | 14W | a | 1 |
| Item6 | 92% | b | 1 |

Gershuny1998

| Item | Description | Judgement | Score |
| --- | --- | --- | --- |
| Item1 | Briefly, 3,156 freshmen at a large midwestern university were first screened with a form of the Short Michigan Alcoholism Screening Test .The current study focused on two waves of data: those collected at Years 1 and 4（N1=489,N2=466）. | b | 1 |
| Item2 | EPQ: neuroticism (a= 0.85), extraversion (a =0 .83), and psychoticism ( a =0 .65); | b | 1 |
| Item3 | Single cohort study, baseline depression levels were measured, but the results were not reported | b | 0 |
| Item4 | BSI: a =0.79 at Year 1, a =0 .76 at Year 4; | a | 1 |
| Item5 | 3Y. Participants were assessed twice over a 3-year interval | a | 1 |
| Item6 | 95% | b | 1 |

Kapci1998

| Item | Description | Judgement | Score |
| --- | --- | --- | --- |
| Item1 | British undergraduate students from different departments of Loughborough University responded to the Beck Depression Inventory (Beck, Rush, Shaw, & Emery, 1979) twice over an interval of seven to ten days. Only the subjects who scored 10 or higher and those who scored 9 or less at both occasions were included in the study. | c | 0 |
| Item2 | 1.Nagative life events  2.Self-worth | b | 1 |
| Item3 | The Time 1 depression was entered first in the regression equation to control for the individual variation in depression scores. | b | 0 |
| Item4 | 2 | a | 1 |
| Item5 | 3M | a | 1 |
| Item6 | 88% | b | 1 |

Cheng1999

| Item | Description | Judgement | Score |
| --- | --- | --- | --- |
| Item1 | Not reported | d | 0 |
| Item2 | The Short Form of the Bem Sex Role Inventory(S-BSRI)， the Inventory of Socially Supportive Behaviors (ISSB) | b | 1 |
| Item3 | Controlled | b | 0 |
| Item4 | Beck Depression Inventory(BDI) | a | 1 |
| Item5 | 6M | a | 1 |
| Item6 | 97% | b | 1 |

Morris1999

| Item | Description | Judgement | Score |
| --- | --- | --- | --- |
| Item1 | First-year undergraduate psychology students | c | 0 |
| Item2 | The Attributional Style Questionnaire (ASQ) of Seligman, Abramson, Semmel, and von Baeyer (1979) | b | 1 |
| Item3 | Thepre-existing level of depression (at Time 1) was statistically  controlled. | b | 0 |
| Item4 | Beck Depression Inventory - Short Form (Beck, 1967) | a | 1 |
| Item5 | 10M | a | 1 |
| Item6 | 68% | c | 0 |

Sakamoto1999

| Item | Description | Judgement | Score |
| --- | --- | --- | --- |
| Item1 | Undergraduates taking an introductory psychology course participated in the first session of the study | c | 0 |
| Item2 | Subscale of Preoccupation Scale ，11 items, | b | 1 |
| Item3 | Difference scores of the SDS (T2SDS  T1SDS) was analyzed | a | 1 |
| Item4 | Zung Self-rating Depression Scale (SDS)，20 items | a | 1 |
| Item5 | 3M | a | 1 |
| Item6 | 60% | c | 0 |

Sakamoto2000

| Item | Description | Judgement | Score |
| --- | --- | --- | --- |
| Item1 | Japanese undergraduates who were taking an introductory psychology course at the University of Tokyo. | c | 0 |
| Item2 | Subscale of Preoccupation Scale, Life Event Questionnaire for undergraduates. | b | 1 |
| Item3 | T1 SDS scores were controlled. | b | 0 |
| Item4 | Zung Self-rating Depression Scale (SDS) | a | 1 |
| Item5 | 4M | a | 1 |
| Item6 | 69% | c | 0 |

Ayduk2001

| Item | Description | Judgement | Score |
| --- | --- | --- | --- |
| Item1 | Women who participated as 1st-year undergraduates in a longitudinal study of dating relationships. The racial composition of the sample was representative of the undergraduate population of the college from which the participants were recruited. | b | 1 |
| Item2 | Rejection Sensitivity Questionnaire (RSQ) (Downey & Feldman, 1996). | b | 1 |
| Item3 | BDI score at Time 1 was included as the covariate. | b | 0 |
| Item4 | Beck Depression Inventory | a | 1 |
| Item5 | 1Y | a | 1 |
| Item6 | Not reported | d | 0 |

Davila2001

| Item | Description | Judgement | Score |
| --- | --- | --- | --- |
| Item1 | The initial sample consisted of 94 male (n = 49) and female (n = 45) UCLA freshmen who were participating in a larger study of early romantic dysfunction and psychopathology. | b | 1 |
| Item2 | DIRI α=0.83；FPAI; | b | 1 |
| Item3 | At T1, 95% of the sample had no symptoms of depression, 2% had mild symptoms, 2% had moderate symptoms, and 1% (one participant) had a diagnosable depression | a | 1 |
| Item4 | BDI; SCID | a | 1 |
| Item5 | 6M | a | 1 |
| Item6 | 93% | b | 1 |

Enns2001

| Item | Description | Judgement | Score |
| --- | --- | --- | --- |
| Item1 | Participation was by anonymous and voluntary response to a mailed survey. | b | 1 |
| Item2 | Multidimensional Perfectionism Scale9 (Hewitt & Flett MPS). ，Multidimensional Perfectionism Scale (Frost MPS)，N and C scales from the NEO Five-Factor Inventory (NEO-FFI) | b | 1 |
| Item3 | Unclear | b | 0 |
| Item4 | Beck Depression Inventory (BDI; 13-item version) | a | 1 |
| Item5 | 6M | a | 1 |
| Item6 | 61%. No significant differences with regard to the following variables: age, gender, BDI score, and maladaptive perfectionism. | b | 1 |

Fresco2001

| Item | Description | Judgement | Score |
| --- | --- | --- | --- |
| Item1 | Seventy-eight undergraduates (54 women participated in this study to fulfill partial course requirements of an introductory psychology class. | c | 0 |
| Item2 | PSI-II（ Personal Style Inventory Ⅱ）, 48 items，sociography : α=0.88， autonomy: α= 0.86 LES（Life experience survey）, 57 items GTA（ Stimulus-Response Inventory of General Trait Anxiousness）, 75items | b | 1 |
| Item3 | Controlling for the respective covariates and main effects. | b | 0 |
| Item4 | Beck Depression Inventory (BDI),21 ITEMS | a | 1 |
| Item5 | 8W | b | 0 |
| Item6 | 94% | b | 1 |

Smith2001

| Item | Description | Judgement | Score |
| --- | --- | --- | --- |
| Item1 | Participants were 35 (11 women, 24 men) students of British and 30 (10 women, 20 men) students of Malaysian nationality1, all full-time students at Cardiff University. The Malaysians were sojourners with plans to return home after completing their programs of study | b | 1 |
| Item2 | Individualism–collectivism scale (INDCOL) , LER（Life events record ） | b | 1 |
| Item3 | Unclear | b | 0 |
| Item4 | BDI（Beck depression inventory ）, 21 items | a | 1 |
| Item5 | 4M | a | 1 |
| Item6 | Unclear | d | 0 |

Scheier2002

| Item | Description | Judgement | Score |
| --- | --- | --- | --- |
| Item1 | Before the start of a fall semester, 1st-year college students who were enrolled at a residential college were recruited to participate in a longitudinal study on adjustment to college, their participation was voluntary | b | 1 |
| Item2 | LOT( Life Orientation Test), α=0.78 Self-Esteem Scale, α=0.87 a modified version of the Cope (Carveretal, 1989), average α=0.74 ISEL( college student version of the Interpersonal Support Evaluation List ) PSS(Perceived Stress Scale ),14-item, T1:α=0.90 T2:α=0.89 | b | 1 |
| Item3 | Unclear | b | 0 |
| Item4 | BDI(Beck Depression Inventory short form ),13-item, T1:α=0.91 T2α=0.84 | a | 1 |
| Item5 | 12-16W | a | 1 |
| Item6 | 90% | b | 1 |

Abela2004

| Item | Description | Judgement | Score |
| --- | --- | --- | --- |
| Item1 | Undergraduates at the University of Pennsylvania. They were recruited from the Psychology Department’s subject pool and received extra credit in their introductory psychology course for participating. | c | 0 |
| Item2 | 1. EASQ consists of a total of 12 hypothetical negative events. Six of the events are of an interpersonal nature. The other six events are of an achievement nature.  2. The CSQ assesses cognitive styles about consequences and the self. To assess the cognitive style about consequences, participants are asked the following question for each of the 12 negative life events. | a | 1 |
| Item3 | The Time 1 BDI score (the covariate) was entered into the equation. By entering this score first, we are controlling for Time 1 depressive symptoms by essentially using our independent variables to predict residual change in BDI scores from Time 1 to Time 2. | a | 1 |
| Item4 | Beck Depression Inventory | a | 1 |
| Item5 | 5W | b | 0 |
| Item6 | 99% | b | 1 |

liu2004

| Item | Description | Judgement | Score |
| --- | --- | --- | --- |
| Item1 | Undergraduate students from two universities in Kumamoto City, Japan | b | 1 |
| Item2 | The Relationship Questionnaire (RQ）Japan version ；one self-made item | b | 1 |
| Item3 | Baseline depression was controlled at the time of analysis | b | 0 |
| Item4 | The Zung Self-Rating Depression Scale (SDS; Zung,1965) | a | 1 |
| Item5 | 3W | b | 0 |
| Item6 | Not reported | d | 0 |

Shahar2004

| Item | Description | Judgement | Score |
| --- | --- | --- | --- |
| Item1 | Participants were 198 Introductory Psychology students at a large southwestern university, who received class credits for participation. | c | 0 |
| Item2 | DEQ（Depressive Experiences Questionnaire ）, DIRI（Depressive Interpersonal Relationship Inventory）, α = 0.89 : NLEQ（ Negative Life Events Questionnaire ）. | b | 1 |
| Item3 | Unclear | b | 0 |
| Item4 | BDI（Beck Depression Inventory）, 21-item，T1:α=0.90 T2:α=0.91 | a | 1 |
| Item5 | 5W | b | 0 |
| Item6 | 96% | b | 1 |

Lindsay2005

| Item | Description | Judgement | Score |
| --- | --- | --- | --- |
| Item1 | Participants of introductory psychology classes | c | 0 |
| Item2 | Goal Orientation Inventory (GOI) | b | 1 |
| Item3 | The relevant BDI-II and RSES change scores between T1 and T2 and T1 and T3as the dependent measures. | a | 1 |
| Item4 | Beck Depression Inventory—II (BDI-II) | a | 1 |
| Item5 | Nearly 3W | b | 0 |
| Item6 | 85% | b | 1 |

Morrison2005

| Item | Description | Judgement | Score |
| --- | --- | --- | --- |
| Item1 | To ensure that we sampled from a relatively high stress period  at time 1 relative to time 2, we ensured that participants were only recruited from classes in which they had degree-related coursework at that time and not at time 2. All study measures were administered to participants from three intact classes. | b | 1 |
| Item2 | RSQ, PSS, IES | a | 1 |
| Item3 | Depression was not measured at T1 | a | 1 |
| Item4 | GHQ, CES-D | a | 1 |
| Item5 | 6M | a | 1 |
| Item6 | 65% | c | 0 |

Wingate2005

| Item | Description | Judgement | Score |
| --- | --- | --- | --- |
| Item1 | Drawn from Introductory Psychology classes at a large state university | c | 0 |
| Item2 | Hopelessness Scale (HS; Beck, Weissman, Lester, Trexler, 1974). | b | 1 |
| Item3 | T1 BDI scores were entered first into the regression equation, thereby creating residual change scores in BDI depressive symptoms from T1 to T2 | a | 1 |
| Item4 | Beck Depression Inventory (BDI; Beck, Rush, Shaw, Emery, 1979; Beck& Steer, 1987) | a | 1 |
| Item5 | 5W | b | 0 |
| Item6 | Not reported | d | 0 |

Joiner2006

| Item | Description | Judgement | Score |
| --- | --- | --- | --- |
| Item1 | Recruited from Introductory Psychology classes at a large state university in the U.S., and received class credit for participation. | c | 0 |
| Item2 | Revised Rosenberg Self–Esteem Questionnaire. | b | 1 |
| Item3 | Time 1 symptom score was inserted into the equation, thereby creating residual change scores in symptoms from Time1 to Time2. | a | 1 |
| Item4 | Beck Depression Inventory | a | 1 |
| Item5 | about 3W | b | 0 |
| Item6 | 97% | b | 1 |

Luhtanen2006

| Item | Description | Judgement | Score |
| --- | --- | --- | --- |
| Item1 | The sample consisted of 795 incoming University of Michigan freshman. Participants were recruited during Freshman Orientation in July and August to “the Adjustment to College Project" | b | 1 |
| Item2 | CSWS(Contingencies of Self–Worth Scale), there’ Approval ：α=0.82, Appearance: Oα=0.82 ,Competition : α=0.89, Academics : α=0.82, Family Support：α=0.82 ,Virtue：α=0.84, God’s Love：α=0.97 | b | 1 |
| Item3 | Based on CES–D norms and recommended cut–scores (Ensel, 1986), 28% of students had scores in the depressed range at time 1 | b | 0 |
| Item4 | CES-D（Center for Epidemiologic Studies Depression Scale ）, α=0.90 | a | 1 |
| Item5 | 6M | a | 1 |
| Item6 | 85% | b | 1 |

Haeffel2007

| Item | Description | Judgement | Score |
| --- | --- | --- | --- |
| Item1 | Participants were unselected undergraduates from the Introductory to Psychology participant pool at the University of Wisconsin-Madison. | c | 0 |
| Item2 | Self-worth IAT, BDI, ALEQ | a | 1 |
| Item3 | The Time 1 depression measure (T1 BDI) was entered in the first step of the regression equation to create a residual change score for the same Time 2 measure (T2 BDI). | a | 1 |
| Item4 | BDI | a | 1 |
| Item5 | 5W | b | 0 |
| Item6 | 96% | b | 1 |

Ito2007

| Item | Description | Judgement | Score |
| --- | --- | --- | --- |
| Item1 | Japanese undergraduate students, taking a human sciences course at a Japanese university participated in the first session of the study (Time 1). They were recruited voluntarily in several class sections of the course. Eight months later, undergraduates from the initial sample, participated in the second session, completing all the measures required (Time 2). | c | 0 |
| Item2 | Response Styles Questionnaire-Japanese version；Negative Rumination Scale; The Zung Self-rating Depression Scale | a | 1 |
| Item3 | Baseline depressive symptoms were statistically controlled | b | 0 |
| Item4 | The Inventory to Diagnose Depression-Legitime Version | a | 1 |
| Item5 | 8M | a | 1 |
| Item6 | 54%. The author claims that "The rest of the sample either dropped out or were absent from class or were eliminated for missing data. There were no significant differences in the measures scores at Time 1 for students who dropped out before Time 2 and those who remained at Time 2." | b | 1 |

Kwon2007

| Item | Description | Judgement | Score |
| --- | --- | --- | --- |
| Item1 | Participants were 305 (62 male, 227 female, 16 not indicated) undergraduate students from a large university in the Northwest. They were recruited from the university’s subject pool and  received course credit for their participation. | b | 1 |
| Item2 | MPS（Multidimensional Perfectionism Scale ）, self-oriented: α=0.87 , other-oriented ：α=0.71 , socially prescribed perfectionism: α=0.80 RSQ（Response Styles Questionnaire）, RSQ-Brood ：α=0.72, RSQ-Reflect: α=0.66 | b | 1 |
| Item3 | Unclear | b | 0 |
| Item4 | BDI(Beck Depression Inventory-II), T1:α=0.87, T2:α=0.89 | a | 1 |
| Item5 | 4W | b | 0 |
| Item6 | 79%. Data from 63 participants were discarded due to their failure to return at Time 2 to complete the second part of the study, and data from an additional 20 participants were discarded due to their failure to follow questionnaire instructions, including not completing entire questionnaires. | b | 1 |

Bjornsson2010

| Item | Description | Judgement | Score |
| --- | --- | --- | --- |
| Item1 | Enrolled in an introductory psychology course, receiving partial course credit | c | 0 |
| Item2 | AAQ,BDI-II, RSQ | a | 1 |
| Item3 | Depression symptoms at T1 as a covariate | b | 0 |
| Item4 | BDI-II | a | 1 |
| Item5 | 8-12W | b | 0 |
| Item6 | 10% | c | 0 |

Haeffel2010

| Item | Description | Judgement | Score |
| --- | --- | --- | --- |
| Item1 | Unselected undergraduates from the University of Wisconsin-Madison; Participants were recruited through a volunteer folder sign-up procedure and were given extra credit points for their participation." | b | 1 |
| Item2 | ALEQ, CSQ | a | 1 |
| Item3 | The Time 1 depression measure (T1 BDI) was entered in the first step of the regression equation to create a residual change score for the same Time 2 measure (T2 BDI). | a | 1 |
| Item4 | BDI | a | 1 |
| Item5 | 5W | b | 0 |
| Item6 | 96% | b | 1 |

Calvete2011

| Item | Description | Judgement | Score |
| --- | --- | --- | --- |
| Item1 | The study was conducted on a sample of first and second year college students at the University of Deusto and the University of the Basque Country, both in Northern Spain Students in science and technical studies represented 34.8% of the sample, and the rest studied social and human sciences | b | 1 |
| Item2 | LSQ(Life Stress Questionnaire), SSQ(Social Stress Questionnaire ), YSQ-SF(Young Schema Questionnaire-Short Form), 75 abandonment:α=0.83 emotional deprivation):α=0.83 defectiveness:α=0.72 dependence:α=0.59 vulnerability to harm: α=：0.72 failure: α=0.84 | b | 1 |
| Item3 | In the first step, depressive symptoms at T1 were introduced as predictors | b | 0 |
| Item4 | CES-D（Center for Epidemiological Studies Depression Scale），20statement s T1α=0.90 T2α=0.88 | a | 1 |
| Item5 | 5M | a | 1 |
| Item6 | 73%. Comparing those participants who were lost to those who remained, the only significant difference between them was the number of stressful events experienced being higher on the lost sample. | b | 1 |

Chang2011

| Item | Description | Judgement | Score |
| --- | --- | --- | --- |
| Item1 | All participants were enrolled in a psychology course and received extra credit for participation. | c | 0 |
| Item2 | PSS, | b | 1 |
| Item3 | Unclear | b | 0 |
| Item4 | BDI | a | 1 |
| Item5 | 2M | b | 0 |
| Item6 | 95% | b | 1 |

Haeffel2011

| Item | Description | Judgement | Score |
| --- | --- | --- | --- |
| Item1 | 131 unselected undergraduates from the volunteer psychology participant pool at the University of Notre Dame. Participants were recruited through an on-line sign-up procedure and were given extra credit points for their participation. | b | 1 |
| Item2 | ALEQ, CSQ, BDI | a | 1 |
| Item3 | The Time 1 depression score (T1 BDI) was entered in the first step of the regression equation to create a residual change score for the same measure at Time 2 (T2 BDI). | a | 1 |
| Item4 | BDI | a | 1 |
| Item5 | 4W | b | 0 |
| Item6 | 97% | b | 1 |

Zou2011

| Item | Description | Judgement | Score |
| --- | --- | --- | --- |
| Item1 | For the first measurement (T1), the second and third graders of School of Finance and economics of Hunan University and Central South University were selected by cluster. The school shall organize and adopt the principle of voluntariness, and sign a voluntary agreement before participating | b | 1 |
| Item2 | General Social and Academic Hassles Scale GASHS，Social support rating scale SSS, Attachment Style Questionnaire ASQ | b | 1 |
| Item3 | Unclear | b | 0 |
| Item4 | Center for Epidemiological Studies Depression Scale ，CES-D | a | 1 |
| Item5 | 1M | b | 0 |
| Item6 | 93% | b | 1 |

Boujut2012

| Item | Description | Judgement | Score |
| --- | --- | --- | --- |
| Item1 | An initial series of interviews was conducted with 40 first year university students. Subsequently, a questionnaire was administered to 1100 additional students | b | 1 |
| Item2 | 33 item coping questionnaire (the Students’ Coping Scale). | b | 1 |
| Item3 | Not reported | b | 0 |
| Item4 | Beck Depression Inventory-Short Form | a | 1 |
| Item5 | 6M | a | 1 |
| Item6 | 51% | c | 0 |

Goldring2012

| Item | Description | Judgement | Score |
| --- | --- | --- | --- |
| Item1 | Chosen by random selection from the Stanford Facebook from the 2006-2007 academic year. | a | 1 |
| Item2 | Self-made scale | b | 1 |
| Item3 | Unclear | b | 0 |
| Item4 | Self-made scale from the Add Health questionnaire and CES-D | a | 1 |
| Item5 | 1W/T, 11T | b | 0 |
| Item6 | 97% | b | 1 |

Huang2012

| Item | Description | Judgement | Score |
| --- | --- | --- | --- |
| Item1 | 261 freshmen were selected | b | 1 |
| Item2 | Depression scale | b | 1 |
| Item3 | Baseline depression was established | a | 1 |
| Item4 | Depression scale | a | 1 |
| Item5 | 4M | a | 1 |
| Item6 | Not reported | d | 0 |

Reilly2012

| Item | Description | Judgement | Score |
| --- | --- | --- | --- |
| Item1 | Recruited from introductory psychology classes at Kent State and Vanderbilt Universities. Students signed up to participate in a study of “College Students’ Thoughts and Feelings” involving a one-time completion of questionnaires in the laboratory. | c | 0 |
| Item2 | CTI, RSE, DAS, ASQ | a | 1 |
| Item3 | Controlling T1 depression | b | 0 |
| Item4 | BDI, CES-D | a | 1 |
| Item5 | 6W | b | 0 |
| Item6 | 36% | c | 0 |

Consedine2013

| Item | Description | Judgement | Score |
| --- | --- | --- | --- |
| Item1 | Survey data for the present study were collected at two time points during students’ first year of college at a small, private university in the northeast of the United States. | b | 1 |
| Item2 | PANSE scale ,24 items, positive social exchanges: T1:α=0.91 T2:α=0.94 negative social exchanges:T1:α=0.84 T2:α=0.94 R-UCLA(revised University of California, Los Angeles (UCLA) Loneliness Scale ), 20-item, T1α=0.91，T2α=0.93 Satisfaction with Life Scale (SWLS), and the positive affect subscale from the CES-D. | b | 1 |
| Item3 | Control variables and time 1 variables are entered in the first step. | b | 0 |
| Item4 | Two subscales from the 20-item CES-D, T1:α=0.85 T2:α=0.87 | a | 1 |
| Item5 | 8M | a | 1 |
| Item6 | 37% | c | 0 |

Hasegawa2013

| Item | Description | Judgement | Score |
| --- | --- | --- | --- |
| Item1 | 437 Undergraduate students (241 women) at the Nagoya University, Tokai Gakuin University, and University of the Ryukyus. Participants were recruited in their classes, mainly psychology classes. | c | 0 |
| Item2 | RRS, CES-D, IDDL | a | 1 |
| Item3 | In multiple regression step 1, Time 1 depression was entered as a predictor | b | 0 |
| Item4 | RRS, CES-D, IDDL | a | 1 |
| Item5 | 8 W. The 8-wk. interval was adopted so that the study could be completed in one semester for practical reasons to minimize attrition. | a | 1 |
| Item6 | 87% | b | 1 |

Sun2013

| Item | Description | Judgement | Score |
| --- | --- | --- | --- |
| Item1 | Taking Anyi, bengyi and Wanyi as the research sites, the first survey (T1) was conducted on all college students of grades 1-2 in three schools, and a total of 11270 students were investigated; One week after the first test, 5% college students were randomly selected from the three schools for 100% retest to test their retest reliability. In addition, after the completion of the first survey, the tracking survey shall be conducted every six months. The total longitudinal tracking time is one year, and the tracking survey shall be conducted twice (T2 and T3). | b | 1 |
| Item2 | Beck The Beck Depression Inventory, BAI Beck Anxiety Inventory， APGAR，Quality Of Life scale QOL，Social Support rate Scale SSS， | b | 1 |
| Item3 | The detection rates of depression were T1： 16.8%， T2： 14.7% and T3： 10.3% The detection rates of depression were T1： 16.8%， T2： 14.7%and T3： 10.3% | b | 0 |
| Item4 | BDI The Beck Depression Inventory | a | 1 |
| Item5 | 1Y | a | 1 |
| Item6 | 90% | b | 1 |

Takagishi2013

| Item | Description | Judgement | Score |
| --- | --- | --- | --- |
| Item1 | Participants were recruited from a nursing university in Kumamoto, Japan. | c | 0 |
| Item2 | CISS, ZSDS | a | 1 |
| Item3 | Unclear | b | 0 |
| Item4 | ZSDS | a | 1 |
| Item5 | An interval of three weeks between each survey. | a | 1 |
| Item6 | 48% | c | 0 |

Zhang2013

| Item | Description | Judgement | Score |
| --- | --- | --- | --- |
| Item1 | A total of 236 questionnaires were distributed and 206 valid questionnaires were recovered, with a recovery rate of 87.29% | c | 0 |
| Item2 | General scale with good reliability and validity | a | 1 |
| Item3 | Not applicable | b | 0 |
| Item4 | BDI | a | 1 |
| Item5 | 4month | a | 1 |
| Item6 | 100% | a | 1 |

Campos2014

| Item | Description | Judgement | Score |
| --- | --- | --- | --- |
| Item1 | A sample of 101 female students who were enrolled in a psychology course | c | 0 |
| Item2 | DEQ、ROD | b | 1 |
| Item3 | Single cohort study, not reported baseline measurements | b | 0 |
| Item4 | The Center for Epidemiologic Studies of Depression Scale (CES-D) | a | 1 |
| Item5 | 6M | a | 1 |
| Item6 | 64.2%. The 45 excluded participants (49 minus the four initially excluded because of invalid DEQ or CES-D protocols) did not significantly differ from the final sample of 52 in age or in their T1 Neediness, Connectedness, Self-Criticism, and Orality scores. | b | 1 |

He2014

| Item | Description | Judgement | Score |
| --- | --- | --- | --- |
| Item1 | 1427 college students were randomly selected from 9 universities in Hangzhou, and 1214 valid questionnaires were representative | a | 1 |
| Item2 | Patient Health Questionnaire-9（PHQ-9），12-Item Short-Form Health Survey （SF-12） ，Adolescent Self-Rating Life Events Check List ( ASLEC) ，Automatic thinking questionnaire (ATQ) ，Self-compiled general demographic data questionnaire | b | 1 |
| Item3 | The average score of PHQ-9 of college students in the preliminary test was (5.56 ± 3.61). 153 students with depressive disorder were screened, accounting for 12.6% of the respondents | b | 0 |
| Item4 | PHQ-9 | a | 1 |
| Item5 | 6M | a | 1 |
| Item6 | 54% | c | 0 |

Ling2014

| Item | Description | Judgement | Score |
| --- | --- | --- | --- |
| Item1 | Using convenient sampling, 900 college students from two universities in Changsha were selected to participate in this study, and 824 valid questionnaires were obtained | b | 1 |
| Item2 | Mood and Anxiety Symptom Questionnaire MASQ-SF，General Social and Academic Hassles Scale GSAHS,Cognitive emotion regulation questionnaire CERQ | b | 1 |
| Item3 | In the second step, considering the high comorbidity rate of anxiety and depression, the baseline levels of depression and anxiety were controlled | b | 0 |
| Item4 | CES-D | a | 1 |
| Item5 | 6W | b | 0 |
| Item6 | Not reported | d | 0 |

Pfeifer2014

| Item | Description | Judgement | Score |
| --- | --- | --- | --- |
| Item1 | Participants, as part of a different unpublished study, completed baseline measures 2 years before the tragic death of the Notre Dame student. | d | 0 |
| Item2 | CSQ, RSS, BDI | a | 1 |
| Item3 | The baseline depression measure was entered in the first step of the regression equation to control for initial levels of depression 2 years before the tragedy. | b | 0 |
| Item4 | BDI | a | 1 |
| Item5 | 2Y | a | 1 |
| Item6 | 41% | c | 0 |

Vanderhasselt2014

| Item | Description | Judgement | Score |
| --- | --- | --- | --- |
| Item1 | Unselected; recruited via the university website | b | 1 |
| Item2 | DAS-A, CERQ | a | 1 |
| Item3 | Unclear | b | 0 |
| Item4 | BDI | a | 1 |
| Item5 | About 3M | a | 1 |
| Item6 | 97% | b | 1 |

Zheng2014

| Item | Description | Judgement | Score |
| --- | --- | --- | --- |
| Item1 | 1.Participants were recruited at two universities in Hunan, China. 2.The sample consisted of 39.3% freshmen, 36.6% sophomores, and 24.1% juniors. In terms of ethnic identity, most participants were Han (91.2%); 6.9% reported “other.” | b | 1 |
| Item2 | SEQ，Rosenberg Self-Esteem Questionnaire，α= 0.77 SHS(General Social and Academic Hassles Scale),α=0.91 | b | 1 |
| Item3 | Unclear | b | 0 |
| Item4 | CES-D(Center for Epidemiological Studies Depression Scale),α=0.88 to 0.91 | a | 1 |
| Item5 | 6M | a | 1 |
| Item6 | 100% | a | 1 |

Levens2016

| Item | Description | Judgement | Score |
| --- | --- | --- | --- |
| Item1 | All participants were freshman undergraduate students in their first year of college that were recruited as part of a larger multi-time point study investigating adjustment to college life. Transfer students were excluded from the study. | b | 1 |
| Item2 | A subset of the Assessment of Social Connectedness (ASC), a well-validated 72-item scale that assesses five types of support: family, friends, neighbors, community members, and others； Perceived Stress Reactivity Scale -PSRS | b | 1 |
| Item3 | Controlled T1depression | b | 0 |
| Item4 | Centre for Epidemiological Studies Depression Scale (CES-D) | a | 1 |
| Item5 | On average 88 (SD = 73) days been sessions (approximately 3  months; range, 20 to 381D) | b | 0 |
| Item6 | 98% | b | 1 |

McGinley2016

| Item | Description | Judgement | Score |
| --- | --- | --- | --- |
| Item1 | The present study utilized data from Wave 1 (W1) and Wave 2 (W2) of a larger study. Study participants were recruited via e-mail from a sample of 9,100 incoming freshmen at eight colleges and universities in the Midwestern United States. | b | 1 |
| Item2 | A modified version of the widely used Sexual Experiences Questionnaire, 13items, T1:men α=0.83, women α=0.87 T2 men α=0.85, women α=0.87 anger/hostility subscale of the Profile of Mood States, 12 items, T1:men α=0.91, women α=0.90 T2 men α=0.90, women α=0.91 RAPI（the Rutgers Alcohol Problems Index）23 items α at W1 = .91 (men and women); α at W2 = .90 (men) and .93 (women) “How often in the last 4 months have you had 4 (for women) or 5 (for men) drinks in a single sitting?” Response options were 0 (Never) to 5 (5 times a week or more). | b | 1 |
| Item3 | Unclear | b | 0 |
| Item4 | CESD（Center for Epidemiologic Studies Depression Scale ）7items，T1:men α=0.84，women α=0.83 T2men α=0.88，women α=0.88 | a | 1 |
| Item5 | Not reported | b | 0 |
| Item6 | Unclear | d | 0 |

Reid2016

| Item | Description | Judgement | Score |
| --- | --- | --- | --- |
| Item1 | First-year, first-semester college students (N = 6,988) from four large United States universities were sent recruitment emails to participate in a web-based self-report survey in fall of 2012. The final fall sample was 1,474 | b | 1 |
| Item2 | CBVS(California Bully-Victimization Scale ), GAD-7(Generalized Anxiety Disorder-7 ),T1α=0.90，T2α=0.91, MSPSS（Multidimensional Scale of Perceived Social Support）, α=0.94 | b | 1 |
| Item3 | In analyses controlling for fall depression | b | 0 |
| Item4 | PHQ-9（Patient Health Questionnaire-9 ）, T1:α=0.88 T2: α=0.89 | a | 1 |
| Item5 | About 6M | a | 1 |
| Item6 | 30% | c | 0 |

Vanderhasselt2016

| Item | Description | Judgement | Score |
| --- | --- | --- | --- |
| Item1 | An unselected group of undergraduate students. Participants were recruited via the university website and received a financial compensation for their participation. | a | 1 |
| Item2 | RRS, AEQ | a | 1 |
| Item3 | Baseline depressive symptoms were statistically controlled | b | 0 |
| Item4 | BDI-II | a | 1 |
| Item5 | 15M | a | 1 |
| Item6 | Unclear | d | 0 |

Junkins2017

| Item | Description | Judgement | Score |
| --- | --- | --- | --- |
| Item1 | 216 freshmen were randomly selected from the university’s freshmen directory | a | 1 |
| Item2 | RRS, BDI, ALEQ | a | 1 |
| Item3 | In multiple regression step 1, Time 1 BDI was entered as a control | b | 0 |
| Item4 | RRS, BDI, ALEQ | a | 1 |
| Item5 | Study 1: 1 month, study 2: 6 months | a | 1 |
| Item6 | Study 1:96%, Study 2:94% | b | 1 |

Richardson2017

| Item | Description | Judgement | | Score | |  |
| --- | --- | --- | --- | --- | --- | --- |
| Item1 | First year British undergraduates recruited from across the UK via students unions. International students were excluded. Ethnicity was 89.6 % (n=405) white. | b | | 1 | |  |
| Item2 | Three-Item Loneliness Scale (Hughes et al., 2004) | b | | 1 | |  |
| Item3 | Unclear | b | | 0 | |  |
| Item4 | Centre for Epidemiological Studies Depression Scale (CES-D) | a | | 1 | |  |
| Item5 | 12-14M | a | | 1 | |  |
| Item6 | 54% | c | | 0 | |  |
| Kate 2018 |  | |  | |  | |
| Items | Quote | | Judgement | | Score | |
| Item 1 | Participants made up 26% of the incoming female class and were representative of the university’s female student body with regard to race/ethnicity | | c | | 0 | |
| Item 2 | Sexual Experiences Survey-Revised (SES-R) | | b | | 1 | |
| Item 3 | Controlling for baseline depression and prior assault history, women who experienced first-semester sexual assault were approximately 2.5 times more likely to have meaningful depression symptoms (OR = 2.45) compared to women who were not assaulted in their first semester (Table 1). | | a | | 1 | |
| Item 4 | Patient Health Questionnaire-9 (PHQ-9) | | a | | 1 | |
| Item 5 | 4 months | | a | | 1 | |
| Item 6 | 87% | | b | | 1 | |
| Cheung2000 |  | |  | |  | |
| Item | Quote | | Judgement | | Score | |
| Item1 | Participants were 154 Mainland Chinese female university students residing in Hong Kong | | c | | 0 | |
| Item2 | MASI,SSS-S | | b | | 1 | |
| Item3 | control for baseline depression | | a | | 1 | |
| Item4 | PHQ-9 | | a | | 1 | |
| Item5 | 8M | | b | | 0 | |
| Item6 | 86% | | b | | 1 | |
| Wang 2020 |  | |  | |  | |
| Items | Quote | | Judgement | | Score | |
| Item1 | Voluntary participation | | b | | 0 | |
| Item2 | Risk factor use scale | | b | | 1 | |
| Item3 | Baseline depressed population was not excluded | | b | | 0 | |
| Item4 | Outcome event Depression Scale | | a | | 1 | |
| Item5 | The follow-up time was 2 months | | b | | 0 | |
| Item6 | 100% | | a | | 1 | |
| Wu2020 |  | |  | |  | |
| Item | Quote | | Judgement | | Score | |
| Item1 | Students at Nanjing University | | b | | 1 | |
| Item2 | 1.RS-11 2. DASS-21 3.PMHS | | b | | 1 | |
| Item3 | control for baseline depression | | a | | 1 | |
| Item4 | DASS-21 | | a | | 1 | |
| Item5 | 3y | | a | | 1 | |
| Item6 | 47% | | c | | 0 | |
| Samek2022 |  | |  | |  | |
| Item | Quote | | Judgement | | Score | |
| Item1 | Seven hundred students were randomly selected out of the entire list of fifirst‐year students, 511 of the eligible 700 were located, and 209 of the 511 completed informed consent and the baseline survey.Participants were generally representative of the population they were sampled from, although they were slightly more likely to be female.. | | b | | 1 | |
| Item2 | Multidimensional Personality Questionnaire | | b | | 1 | |
| Item3 | control for baseline depression | | a | | 1 | |
| Item4 | CES-D | | a | | 1 | |
| Item5 | 4y | | a | | 1 | |
| Item6 | 80%,There were no signifificant difffferences in participation rates at Wave 2 or 3 based on demographics and AUD or depressive symptoms. | | b | | 1 | |

**Supporting information S4-2. Literature quality evaluation of retrospective cohort studies**

Katz2009

| Items | Description | Judgement | Score |
| --- | --- | --- | --- |
| Item 1 | Undergraduate women growing up in an intact family with one mother and one father. | c | 0 |
| Item 2 | The Relationship with Parents Scale (RPS; Alexander, 2003)；Adult Attachment Scale (AAS) ；four items developed by Joiner et al. | b | 1 |
| Item 3 | No | b | 0 |
| Item 4 | Centre for Epidemiological Studies Depression Scale (CES-D) | a | 1 |
| Item 5 | More than 1 year | a | 1 |
| Item 6 | complete follow up | a | 1 |

Stolow2012

| Items | Description | Judgement | Score |
| --- | --- | --- | --- |
| Item 1 | Participants were recruited in two phases. In the first phase, participants were recruited through an upper-level psychology course and received course credit for their voluntary participation in the study. In the second phase, recruitment posters were distributed around Montreal-area universities | c | 0 |
| Item 2 | CSQ (The Cognitive Style Questionnaire), LSI (The Life Story Interview), | b | 1 |
| Item 3 | No | b | 0 |
| Item 3 | BDI(The Beck Depression Inventory)，α=0.89 | a | 1 |
| Item 4 | More than 1 year | a | 1 |
| Item 5 | complete follow up | a | 1 |

Zhong2014

| Items | Description | Judgement | Score |
| --- | --- | --- | --- |
| Item 1 | Seven universities in Harbin were selected to issue questionnaires | b | 1 |
| Item 2 | Childhood Trauma Questionnaire CTQ-SF, Close Relationships Questionnaire RQ, College and Secondary school Students Life Event Scale CSS-LES、ATQ | b | 1 |
| Item 3 | No | b | 0 |
| Item 4 | CES-D | a | 1 |
| Item 5 | More than 3 years | a | 1 |
| Item 6 | complete follow up | a | 1 |

He2016

| Items | Description | Judgement | Score |
| --- | --- | --- | --- |
| Item 1 | Random sampling. Freshmen to junior students in a university in Yantai | a | 1 |
| Item 2 | Childhood trauma experience rating scale, Chinese depression sensitive personality questionnaire | b | 1 |
| Item 3 | No | b | 0 |
| Item 4 | SDS | a | 1 |
| Item 5 | More than 3 years | a | 1 |
| Item 6 | complete follow up | a | 1 |

Adjorlolo2017

| Items | Description | Judgement | 得分 |
| --- | --- | --- | --- |
| Item 1 | Data were collected from 300 undergraduate students in the University of Ghana main campus in 2014 | b | 1 |
| Item2 | Childhood Trauma Questionnaire,25 items. Physical abuse: α =0 .74, emotional abuse: α =0 .79, sexual abuse: α =0 .66, physical neglect: α =0.55 emotional neglect: α =0 .84, General Self-Efficacy Scale (GSE), 8-item, α=0.94, Satisfaction with Life Scale (SWLS), 5 items, α=0.76 | b | 1 |
| Item 3 | No | b | 0 |
| Item 4 | Beck’s Depression Inventory-II (BDI-II) , 21 items，α=0.73 | a | 1 |
| Item 5 | More than 3 years | a | 1 |
| Item 6 | complete follow up | a | 1 |

Hu2018

| Items | Description | Judgement | Score |
| --- | --- | --- | --- |
| Item1 | 275 medical college students were selected by stratified random sampling. 262 valid questionnaires were collected after on-site evaluation | c | 0 |
| Item2 | Personal Report of Childhood Abuse（PRCA）, Trait coping style questionnaire（TCSQ）, Self-Rating Anxiety Scale (SAS), suicide ideation scale（SIS）, Self-made questionnaire | b | 1 |
| Item 3 | No | b | 0 |
| Item 4 | BDI | a | 1 |
| Item 5 | More than 3 years | a | 1 |
| Item 6 | complete follow up | a | 1 |

Li 2018

| Items | Quote | Judgement | Score |
| --- | --- | --- | --- |
| Item 1 | random sampling | b | 1 |
| Item 2 | risk factors use scale | b | 1 |
| Item 3 | No | b | 0 |
| Item 4 | outcome event use depression scale | a | 1 |
| Item 5 | More than 3 years | a | 1 |
| Item 6 | complete follow up | a | 1 |

Rong 2018

| Items | Description | Judgement | Score |
| --- | --- | --- | --- |
| Item 1 | Cluster sampling | b | 1 |
| Item 2 | Childhood Trauma Questionnaire | b | 1 |
| Item 3 | No | b | 0 |
| Item 4 | Self-Rating Depression Scale | a | 1 |
| Item 5 | More than 3 years | a | 1 |
| Item 6 | complete follow up | a | 1 |

Yang 2018

| Items | Quote | Judgement | Score |
| --- | --- | --- | --- |
| Item 1 | cluster sampling | b | 1 |
| Item 2 | risk factors use scale | b | 1 |
| Item 3 | No | b | 0 |
| Item 4 | outcome event use depression scale | a | 1 |
| Item 5 | More than 3 years | a | 1 |
| Item 6 | complete follow up | a | 1 |

Fasciano2019

| Items | quote | Judgement | score |
| --- | --- | --- | --- |
| Item 1 | 90 undergraduates: Caucasian (65.6%), while other participants reported being African American (20.0%), other (7.8%), Asian/Pacific Islander (4.4%), and Multicultural (2.2%) | b | 1 |
| Item 2 | 1.CTQ 2. MAQA 3.SES 4.PCL-C | b | 1 |
| Item 3 | No | b | 0 |
| Item 4 | corresponding subscales of the Brief Symptom Inventory (BSI) | a | 1 |
| Item 5 | More than 3 years | a | 1 |
| Item 6 | complete follow up | a | 1 |

Zhao2019

| Items | Quote | Judgement | Score |
| --- | --- | --- | --- |
| Item 1 | random number sampling | b | 1 |
| Item 2 | risk factors use scale | b | 1 |
| Item 3 | No | b | 0 |
| Item 4 | outcome event use depression scale | a | 1 |
| Item 5 | More than 3 years | a | 1 |
| Item 6 | complete follow up | a | 1 |

Ma2020

| Items | Quote | Judgement | Score |
| --- | --- | --- | --- |
| Item 1 | stratified sampling | b | 1 |
| Item 2 | risk factors use scale | b | 1 |
| Item 3 | No | b | 0 |
| Item 4 | outcome event use depression scale | a | 1 |
| Item 5 | More than 3 years | a | 1 |
| Item 6 | complete follow up | a | 1 |

Wang2020

| Items | quote | Judgement | score |
| --- | --- | --- | --- |
| Item 1 | Normative college students | b | 1 |
| Item 2 | 1.CTQ-SF 2.CD-RISC 3.NEO-FFI | b | 1 |
| Item 3 | No | b | 0 |
| Item 4 | BDI | a | 1 |
| Item 4 | More than 3 years | a | 1 |
| Item 6 | complete follow up | a | 1 |

Xia2020

| Items | Quote | Judgement | Score |
| --- | --- | --- | --- |
| Item 1 | cluster sampling | b | 1 |
| Item 2 | risk factors use scale | b | 1 |
| Item 3 | No | b | 0 |
| Item 4 | outcome event use depression scale | a | 1 |
| Item 5 | More than 3 years | a | 1 |
| Item 6 | complete follow up | a | 1 |
