# Supplementary material for: Influencing factors of depressive symptoms among undergraduates: A systematic review and meta-analysis
Source: PLoS One. 2023 Mar 2;18(3):e0279050. doi: 10.1371/journal.pone.0279050 (PMC9980735; doi:10.1371/journal.pone.0279050)
Supplement: S1 File — (DOCX) [file pone.0279050.s006.docx]

**Supporting information S1 File. Search strategy of the research**

**Embase (Ovid)**

1. exp Depression/

2. exp Depressive Disorder/

3. (Unipolar Depressions or Depressions, Unipolar or Depression, Unipolar or Unipolar Depression or Melancholias or Melancholia or Neurotic Depressions or Neurotic Depression or Depressions, Neurotic or Depression, Neurotic or Syndromes, Depressive or Syndrome, Depressive or Depressive Syndromes or Depressive Syndrome or Endogenous Depressions or Endogenous Depression or Depressions, Endogenous or Neuroses, Depressive or Depressive Neurosis or Depressive Neuroses or Neurosis, Depressive or Disorders, Depressive or Disorder, Depressive or Depressive Disorders or Emotional Depressions or Depressions, Emotional or Depression, Emotional or Emotional Depression or Symptoms, Depressive or Symptom, Depressive or Depressive Symptoms or Depressive Symptom or Depressions).tw.

4. exp undergraduate student/

5. (undergraduate or undergraduates).tw.

6. exp college student/

7. college students.tw.

8. exp university student/

9. university students.tw.

10. exp case control studies/

11. exp Cohort Studies/

12. exp Longitudinal Studies/

13. exp retrospective studies/

14. exp Follow-Up Studies/

15. exp prospective studies/

16. Cohort.ti,ab.

17. case control.ti,ab.

18. longitudinal.ti,ab.

19. follow up.ti,ab.

20. prospective*.ti,ab.

21. retrospective*.ti,ab.

22. nonrandom*.ti,ab.

23. comparison group*.ti,ab.

24. control group*.ti,ab.

25. exp Registries/

26. registry.ti,ab.

27. registries.ti,ab.

28. randomized controlled trial.pt.

29. Controlled clinical trial.pt.

30. Randomized.ti,ab.

31. Placebo.ti,ab.

32. exp Clinical Trials as Topic/

33. randomly.ti,ab.

34. 1 or 2 or 3

35. 4 or 5 or 6 or 7 or 8 or 9

36. 10 or 11 or 12 or 13 or 14 or 15 or 16 or 17 or 18 or 19 or 20 or 21 or 22 or 23 or 24 or 25 or 26 or 27 or 28 or 29 or 30 or 31 or 32 or 33

37. 34 and 35 and 36

38. limit 37 to yr="2018 -Current"

**Scopus**

#1 ( TITLE-ABS-KEY ( "depression" ) OR TITLE-ABS-KEY ( "depressive disorder" ) OR TITLE-ABS-KEY ( "Depressions" ) OR TITLE-ABS-KEY ( "Depressive Symptoms" ) OR TITLE-ABS-KEY ( "Depressive Symptom" ) OR TITLE-ABS-KEY ( "Symptom, Depressive" ) OR TITLE-ABS-KEY ( "Symptoms, Depressive" ) OR TITLE-ABS-KEY ( "Emotional Depression" ) OR TITLE-ABS-KEY ( "Depression, Emotional" ) OR TITLE-ABS-KEY ( "Depressions, Emotional" ) OR TITLE-ABS-KEY ( "Emotional Depressions" ) OR TITLE-ABS-KEY ( "Depressive Disorders" ) OR TITLE-ABS-KEY ( "Disorder, Depressive" ) OR TITLE-ABS-KEY ( "Disorders, Depressive" ) OR TITLE-ABS-KEY ( "Neurosis, Depressive" ) OR TITLE-ABS-KEY ( "Depressive Neuroses" ) OR TITLE-ABS-KEY ( "Depressive Neurosis" ) OR TITLE-ABS-KEY ( "Neuroses, Depressive" ) OR TITLE-ABS-KEY ( "Depression, Endogenous" ) OR TITLE-ABS-KEY ( "Depressions, Endogenous" ) OR TITLE-ABS-KEY ( "Endogenous Depression" ) OR TITLE-ABS-KEY ( "Endogenous Depressions" ) OR TITLE-ABS-KEY ( "Depressive Syndrome" ) OR TITLE-ABS-KEY ( "Depressive Syndromes" ) OR TITLE-ABS-KEY ( "Syndrome, Depressive" ) OR TITLE-ABS-KEY ( "Syndromes, Depressive" ) OR TITLE-ABS-KEY ( "Depression, Neurotic" ) OR TITLE-ABS-KEY ( "Depressions, Neurotic" ) OR TITLE-ABS-KEY ( "Neurotic Depression" ) OR TITLE-ABS-KEY ( "Neurotic Depressions" ) OR TITLE-ABS-KEY ( melancholia ) OR TITLE-ABS-KEY ( melancholias ) OR TITLE-ABS-KEY ( "Unipolar Depression" ) OR TITLE-ABS-KEY ( "Depression, Unipolar" ) OR TITLE-ABS-KEY ( "Depressions, Unipolar" ) OR TITLE-ABS-KEY ( "Unipolar Depressions" ) )

#2 ( TITLE-ABS-KEY ( "university students" ) OR TITLE-ABS-KEY ( "university student" ) OR TITLE-ABS-KEY ( "college students" ) OR TITLE-ABS-KEY ( "college student" ) OR TITLE-ABS-KEY ( undergraduate ) OR TITLE-ABS-KEY ( undergraduates ) )

#3 ( TITLE-ABS-KEY ( "Randomized controlled trial" ) OR TITLE-ABS-KEY ( "Controlled clinical trial" ) OR TITLE-ABS-KEY ( "Randomized OR Placebo" ) OR TITLE-ABS-KEY ( "Clinical Trials" ) OR TITLE-ABS-KEY ( randomly ) OR TITLE-ABS-KEY ( "case control studies" ) OR TITLE-ABS-KEY ( "Cohort Studies" ) OR TITLE-ABS-KEY ( "Longitudinal Studies" ) OR TITLE-ABS-KEY ( "retrospective studies" ) OR TITLE-ABS-KEY ( "Follow-Up Studies" ) OR TITLE-ABS-KEY ( "prospective studies" ) OR TITLE-ABS-KEY ( cohort ) OR TITLE-ABS-KEY ( "case control" ) OR TITLE-ABS-KEY ( longitudinal ) OR TITLE-ABS-KEY ( "follow up" ) OR TITLE-ABS-KEY ( prospective* ) OR TITLE-ABS-KEY ( retrospective* ) OR TITLE-ABS-KEY ( "comparison group*" ) OR TITLE-ABS-KEY ( "control group*" ) )

#4 #1 and #2 and #3

**Pubmed**

((((("case control studies”[Mesh] OR "Cohort Studies"[Mesh] OR "Longitudinal Studies"[Mesh] OR "retrospective studies” [Mesh] OR "Follow-Up Studies"[ Mesh] OR "prospective studies" [Mesh] OR Cohort[tiab] OR case control[tiab] OR longitudinal[tiab] OR follow up[tiab] OR prospective*[tiab] OR retrospective*[tiab] OR nonrandom*[tiab] OR comparison group*[tiab] OR control group*[tiab] OR "Registries"[Mesh] OR registry[tiab] OR registries[tiab])) OR (Randomized controlled trial [pt] OR Controlled clinical trial [pt] OR Randomized [tiab] OR Placebo [tiab] OR Clinical Trials as Topic [Mesh] OR randomly [tiab] OR Trials [ti]))) AND ((((((undergraduates) OR undergraduate) OR "college student") OR "college students") OR "university student") OR "university students")) AND (((((((((((((((((((((((((((Unipolar Depressions[Text Word]) OR Depressions, Unipolar[Text Word]) OR Depression, Unipolar[Text Word]) OR Unipolar Depression[Text Word]) OR Melancholias[Text Word]) OR Melancholia[Text Word]) OR Neurotic Depressions[Text Word]) OR Neurotic Depression[Text Word]) OR Depressions, Neurotic[Text Word]) OR Depression, Neurotic[Text Word]) OR Syndromes, Depressive[Text Word]) OR Syndrome, Depressive[Text Word]) OR Depressive Syndromes[Text Word]) OR Depressive Syndrome[Text Word]) OR Endogenous Depressions[Text Word]) OR Endogenous Depression[Text Word]) OR Depressions, Endogenous[Text Word]) OR Neuroses, Depressive[Text Word]) OR Depressive Neurosis[Text Word]) OR Depressive Neuroses[Text Word]) OR Neurosis, Depressive[Text Word]) OR Disorders, Depressive[Text Word]) OR Disorder, Depressive[Text Word]) OR Depressive Disorders[Text Word])) OR (((((((((Emotional Depressions[Text Word]) OR Depressions, Emotional[Text Word]) OR Depression, Emotional[Text Word]) OR Emotional Depression[Text Word]) OR Symptoms, Depressive[Text Word]) OR Symptom, Depressive[Text Word]) OR Depressive Symptoms[Text Word]) OR Depressive Symptom[Text Word]) OR Depressions[Text Word])) OR ("Depression"[Mesh] OR "Depressive Disorder"[Mesh]))

**PsycINFO, PsycARTICLES :**

( college students or university students or undergraduates ) AND SU ( depression or depressive disorder or depressive symptoms or major depressive disorder )

**CNKI:**

(SU='抑郁') and (SU='大学' or SU='高校' or SU='高校学生' or SU='大学生')

**Wanfang:**

(主题="抑郁")*(主题=("大学生"+"高校"+"大学"+"高校学生"))

**VIP:**

(M=抑郁) AND (M=大学 OR M=高校 OR M=大学生 OR M=高校学生)
